# Runx1 Transcription Factor Is Required for Myoblasts Proliferation during Muscle Regeneration

**DOI:** 10.1371/journal.pgen.1005457

**Published:** 2015-08-14

**Authors:** Kfir Baruch Umansky, Yael Gruenbaum-Cohen, Michael Tsoory, Ester Feldmesser, Dalia Goldenberg, Ori Brenner, Yoram Groner

**Affiliations:** 1 Department of Molecular Genetics, The Weizmann Institute of Science, Rehovot, Israel; 2 Department of Veterinary Resources, The Weizmann Institute of Science, Rehovot, Israel; 3 Grand Israel National Center for Personalized Medicine (INCPM), The Weizmann Institute of Science, Rehovot, Israel; The Jackson Laboratory, UNITED STATES

## Abstract

Following myonecrosis, muscle satellite cells proliferate, differentiate and fuse, creating new myofibers. The Runx1 transcription factor is not expressed in naïve developing muscle or in adult muscle tissue. However, it is highly expressed in muscles exposed to myopathic damage yet, the role of Runx1 in muscle regeneration is completely unknown. Our study of Runx1 function in the muscle’s response to myonecrosis reveals that this transcription factor is activated and cooperates with the MyoD and AP-1/c-Jun transcription factors to drive the transcription program of muscle regeneration. Mice lacking dystrophin and muscle Runx1 (*mdx* ^*-*^ */Runx1* ^*f/f*^), exhibit impaired muscle regeneration leading to age-dependent muscle waste, gradual decrease in motor capabilities and a shortened lifespan. Runx1-deficient primary myoblasts are arrested at cell cycle G_1_ and consequently differentiate. Such premature differentiation disrupts the myoblasts’ normal proliferation/differentiation balance, reduces the number and size of regenerating myofibers and impairs muscle regeneration. Our combined Runx1-dependent gene expression, ChIP-seq, ATAC-seq and histone H3K4me1/H3K27ac modification analyses revealed a subset of Runx1-regulated genes that are co-occupied by MyoD and c-Jun in *mdx* ^*-*^ */Runx1* ^*f/f*^ muscle. The data provide unique insights into the transcriptional program driving muscle regeneration and implicate Runx1 as an important participant in the pathology of muscle wasting diseases.

## Author Summary

In response to muscle injury, the muscle initiates a repair process that calls for the proliferation of muscle stem cells, which differentiate and fuse to create the myofibers that regenerate the tissue. Maintaining the balance between myoblast proliferation and differentiation is crucial for proper regeneration, with disruption leading to impaired regeneration characteristic of muscle-wasting diseases. Our study highlights the important role the Runx1 transcription factor plays in muscle regeneration and in regulating the balance between muscle stem cell proliferation and differentiation. While not expressed in healthy muscle tissue, Runx1 level significantly increases in response to various types of muscle damage. This aligns with our finding that mice lacking Runx1 in their muscles suffer from impaired muscle regeneration. Their muscles contained a significantly low number of regenerating myofibers, which were also relatively smaller in size, resulting in loss of muscle mass and motor capabilities. Our results indicate that Runx1 regulates muscle regeneration by preventing premature differentiation of proliferating myoblasts, thereby facilitating the buildup of the myoblast pool required for proper regeneration. Through genome-wide gene-expression analysis we identify a set of Runx1-regulated genes responsible for muscle regeneration thereby implicating Runx1 in the pathology of muscle wasting diseases such as Duchenne muscular dystrophy.

## Introduction

Striated muscles are highly organized structure composed of bundles of multinucleated myofibers. Each myofiber harbors peripheral nuclei and highly-organized myofibrils, granting the muscle its contractile force [1]. Muscle satellite cells (SC) comprise 2–5% of adult muscle cells [2]. Located at the myofiber periphery, SC are quiescent, myoblast-committed cells that serve as the muscle’s “stem cell” reservoir. Muscles subjected to regeneration-inducing damage, such as trauma or muscle dystrophy, use this reservoir to create new muscle fibers. Muscle regeneration involves the sequential induction of muscle-specific transcription factors (TFs), including the myogenic regulatory factors (MRFs) *Myf5*, *Myod*, *Myog* and *Mrf4*. Proliferating myoblasts express *Myf5* and *Myod*, whereas *Myog* is induced at the onset of differentiation and drives myoblast terminal differentiation [3]. Yet, the role of Runx1 TF in muscle regeneration remains to be determined.

Runx1 is a member of the RUNX family of TFs, which regulate cell lineage determination in several developmental pathways [4]. While Runx1 is not detected in naïve embryonic developing muscle [5,6] or in adult muscle tissue [7], it is highly expressed in muscles exposed to myopathic damage. *RUNX1* expression was found to be significantly increased in samples of muscle dystrophies, including mouse models of Duchenne muscular dystrophy (DMD) [8] and amyotrophic lateral sclerosis (ALS) [9], myopathy patients (including EDMD, DMD, AQM [10]) and in cardiotoxin (CTX)-treated muscle [11]. Genome-wide ChIP-seq analysis using C2C12 cells revealed enrichment of RUNX and AP-1 motifs at MyoD-bound regions [12]. Runx and AP-1 motifs were also enriched in C2C12 cell MyoD-bound enhancers [13], and several genomic loci co-occupied by MyoD and AP-1 factor c-Jun also bound Runx1 [13]. Based on these findings in C2C12 cells, it was suggested that Runx1, MyoD and c-Jun assemble on the same regulatory regions, to promote myoblasts differentiation. However, other experiments involving myoblastic or transformed cell lines led to conflicting conclusions regarding the role of Runx1 in myoblasts. Inhibition of Runx1 activity in C2C12 either directly or by knockdown of its obligatory cofactor Cbf-β or led to enhanced differentiation [14]. On the other hand, similar enhanced differentiation was observed upon forced expression of Runx1 in rhabdomyosarcoma cells [15]. These data suggested that Runx1 could function as both repressor or activator of myoblast differentiation.

To investigate the function of Runx1 in muscle regeneration in a direct *in vivo* approach, we first generated mice lacking muscle Runx1 (*Runx1* ^*f/f*^). Using these mice we found that Runx1 is switched on in response to muscle damage and participates in muscle regeneration by preventing premature myoblasts differentiation. Moreover, when crossed onto the DMD mouse model (*mdx* mice), the Runx1-deficient *mdx* mice (*mdx/Runx1* ^*f/f*^) encountered defects in muscle mass and muscle strength that are not part of the *mdx* phenotype thereby highlighting the involvement of Runx1 in muscle regeneration. At the cellular level *mdx* ^*-*^ */Runx1* ^*f/f*^ mice showed impaired myoblast proliferation that impeded muscle regeneration and contributed to the severity of muscle deterioration. Genome-wide analyses of *Runx1* ^*f/f*^ primary myoblasts (PM) revealed that PM Runx1 cooperates with MyoD and c-Jun to transcriptionally regulate a subset of genes that prevent premature myoblast differentiation. These data add unique insight on the transcriptional program driving muscle regeneration and implicate Runx1 as an important participant in the pathology of muscle-wasting diseases.

## Results

### Muscle damage-induced expression of Runx1

**Fig 1:**
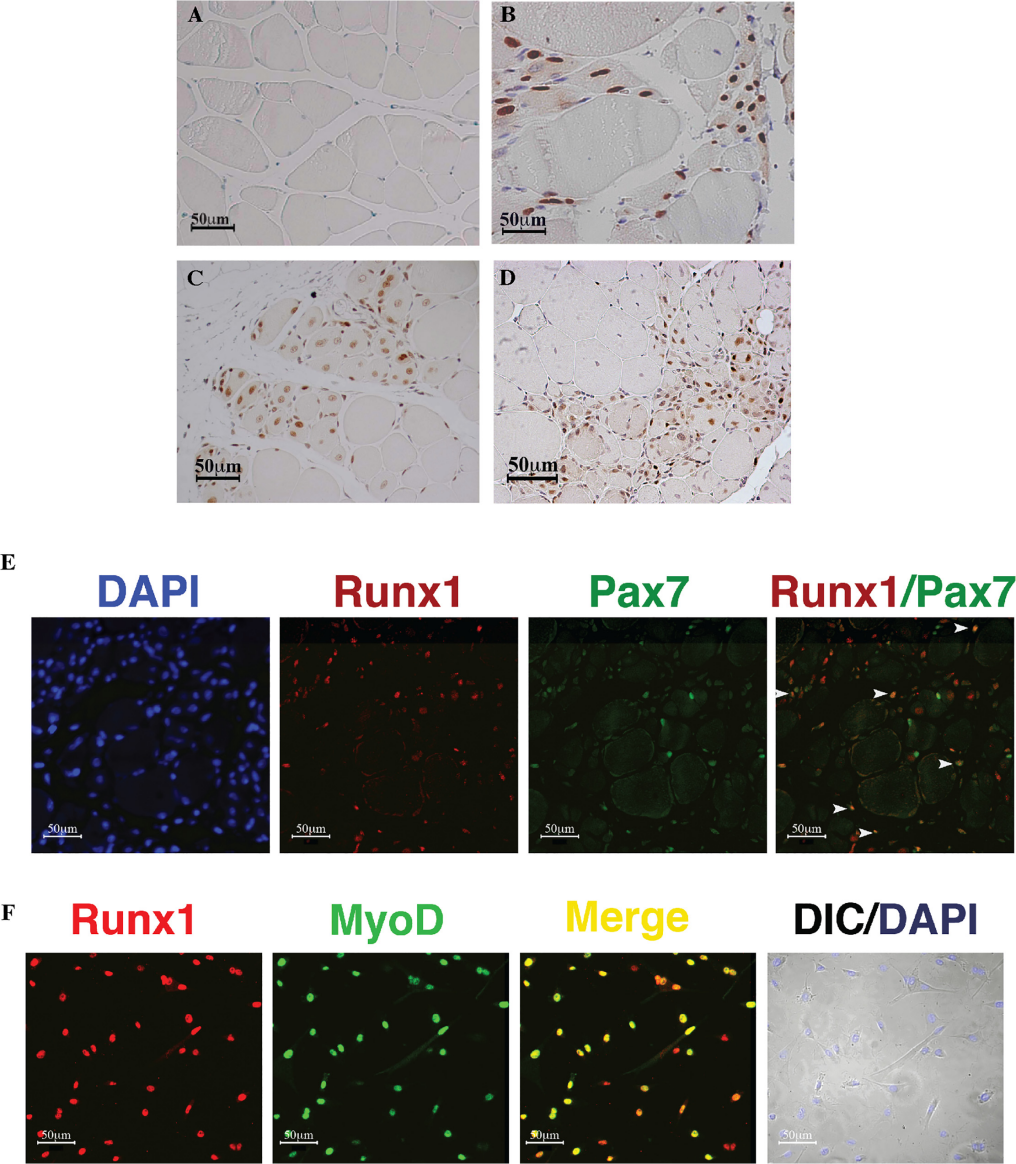
Runx1 expression in response to muscle damage. (A to D). IHC using anti- Runx1 Ab of gastrocnemius muscle from mice subjected to muscle stress. Runx1-positive cells show brown nuclear staining, scale bars, 50 μm. (A) Untreated WT mice. (B) 120 days-old *tg-mSOD1* mice. (C) CTX treated WT mice. (D) 2 month old *mdx* mice. (E) Runx1 and Pax7 IF analysis of CTX-treated WT muscle, scale bars, 50 μm. White arrowheads indicate Runx1^+^/Pax7^+^ cells. (F) IF analysis of cultured proliferating PM using anti- Runx1 and MyoD Abs. DAPI staining was used as a nuclear marker, and myoblasts were visualized by differential interface contrast (DIC) microscopy, scale bars, 50 μm. Results from one of four different experiments with similar findings are shown.

As noted above, *Runx1* RNA expression was reported previously in various types of human muscle diseases including ALS and DMD and their respective mouse models *tg-mSOD1* and *mdx*. Immunohistochemistry (IHC) analysis of gastrocnemius muscles by anti Runx1 antibodies (Ab) revealed no signal in untreated wild-type (WT) muscle (Fig 1A) and in developing muscle (S1A Fig), whereas it was readily detected in *tg-mSOD1* muscles (Fig 1B) and in denervated muscles (see S2 Fig). Significantly, Runx1 was also readily detected in nuclei of regenerating CTX-treated or *mdx* muscles (Fig 1C and 1D). This observation suggests that Runx1 participates in muscle regeneration, an interpretation further supported by the presence of a cell population that co-expressed Runx1 and the SC-expressing TF Pax7 (Fig 1E), indicating that Runx1 is expressed in SC during regeneration. Finally, Runx1 expression was also observed in cultured PM (Figs 1F and S1A). Of note, all the Runx1^+^ cells in these PM cultures also expressed Pax7 (S1B Fig). Thus, muscle Runx1, which is not expressed during development or in resting WT muscle, is activated in response to either neuronal-mediated muscle damage, or myonecrosis.

### Phenotypic features of Runx1-deficient *mdx* mice corroborate the essential role of Runx1 in muscle regeneration

**Fig 2:**
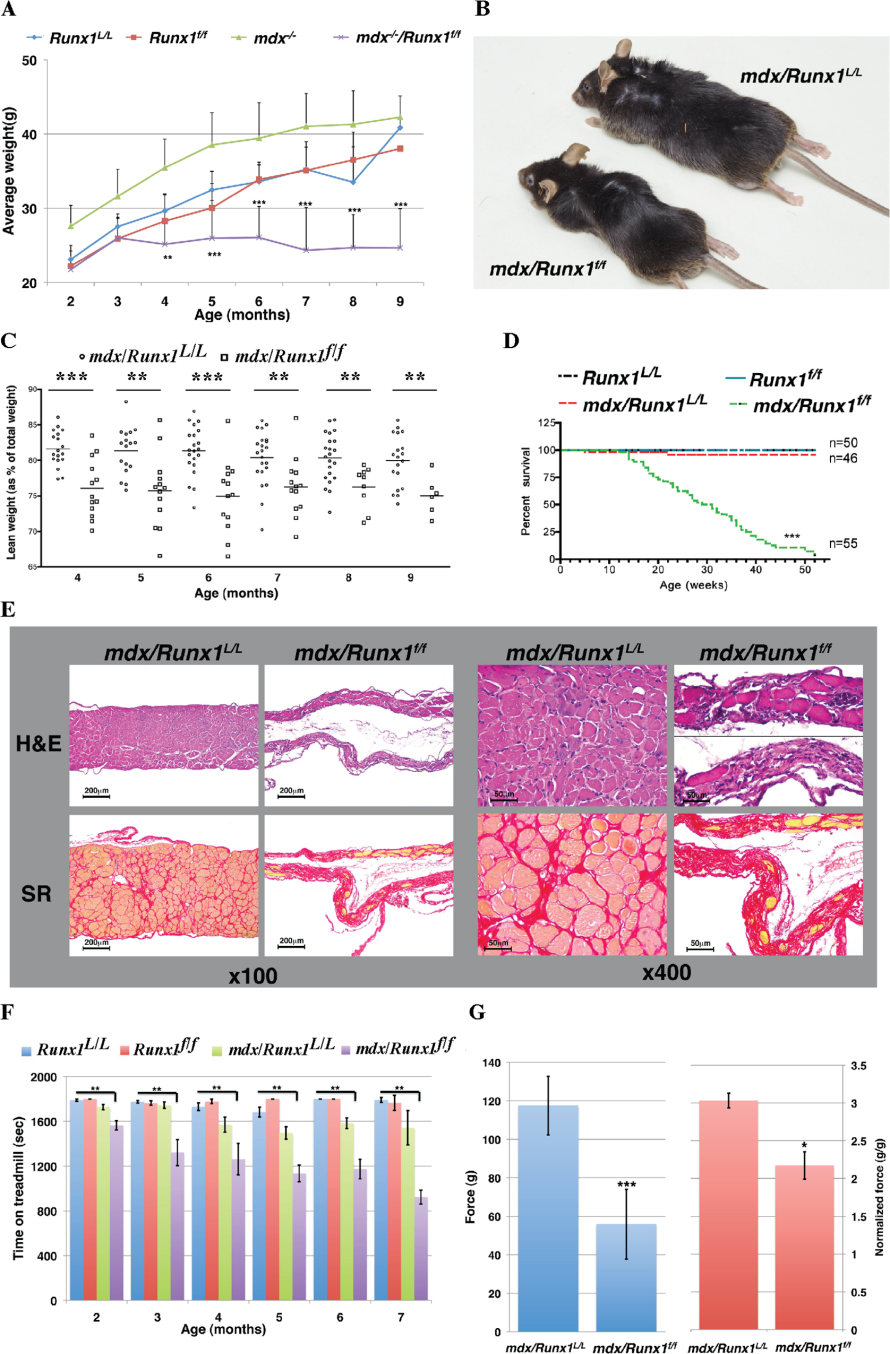
Loss of Runx1 in *mdx* mice decreases muscle mass, muscle strength and lifespan. (A) Scatter plot showing weight of *Runx1* ^*L/L*^ (WT), *Runx1* ^*f/f*^, *mdx* and *mdx/Runx1* ^*f/f*^ mice between 2–9 months of age (average ±SD, n = 9–28, **P<0.01). (B) Representative image of *mdx* and *mdx/Runx1* ^*f/f*^ littermates at 7 months of age. (C) Dot plot depicting the average lean weight (as % of total body weight) of *mdx* and *mdx/Runx1* ^*f/f*^ mice between 4–9 months of age. *mdx* = open circles, and *mdx/Runx1* ^*f/f*^ = open squares. Mean lean weight is indicated (n = 6–22, **P<0.01, ***P<0.0001). (D) Kaplan- Meyer survival curve of *Runx1* ^*L/L*^ (n = 50, blue), *Runx1* ^*f/f*^ (n = 50, red), *mdx* (n = 46, green) and *mdx/Runx1* ^*f/f*^ (n = 55, purple) (***P<0.0001). (E) Diaphragm muscle sections of *mdx* and *mdx/Runx1* ^*f/f*^ mice stained with H&E (top) or Sirius Red (bottom) for collagen (Fibrosis), shown at x100 (left panels) or x400 (right panels). Scale bars, 200μm and 50μm for the X100 and X400 magnifications, respectively. (F) Histogram summarizing treadmill performance of mice between 2–7 months of age. *Runx1* ^*L/L*^, *Runx1* ^*f/f*^, *mdx* and *mdx/Runx1* ^*f/f*^ mice were subjected to an exhaustion protocol (Average ±SD, n = 5–21, **P<0.01, Bonferroni corrected post-hoc comparisons). (G) 4 months old *mdx* and *mdx/Runx1* ^*f/f*^ mice were subjected to grip strength measurements. Left and right histograms show absolute and normalized (to body weight) force comparison respectively. Values are mean ±SEM (n = 9–14 *P<0.05, **P<0.01).

To elucidate Runx1 function during muscle regeneration, we first created mice lacking muscle Runx1 by crossing *Runx1* ^*LoxP/LoxP*^ (*Runx1* ^*L/L*^) mice [16] onto transgenic *Myf5*::*Cre* mice that express Cre in early muscle development and regeneration [17] (S2A Fig, left panel, *Runx1* ^*f/f*^). As *Runx1* expression was previously reported to be elevated in denervated muscle [7], we determined the levels of muscle specific Runx1 mRNA (S2B and S2C Fig) and protein (S2D Fig) in denervated muscle and thymus of *Runx1* ^*f/f*^ mice compared to WT *Runx1* ^*L/L*^ mice. Runx1 RNA and protein levels were elevated in the denervated WT muscle, yet its levels did not change upon denervation of the *Runx1* ^*f/f*^ muscle (S2B–S2D Fig). No significant differences were observed in thymi of *Runx1* ^*L/L*^ or *Runx1* ^*f/f*^ mice (S2B–S2D Fig). Of note, while *Myf5*::*Cre* is active from early stages of muscle development, loss of Runx1 is actually confined to fibers responding to muscle damage. Indeed, body weight and myofiber size of *Runx1* ^*f/f*^ and *Runx1* ^*L/L*^ littermate mice were similar (S2E and S2F Fig). The muscle specific Runx1-deficient mice (*Runx1* ^*f/f*^) were then crossed onto an *mdx* mice to generate *mdx* mice lacking muscle Runx1 (S2A Fig, right panel, *mdx/Runx1* ^*f/f*^). Muscle specific ablation of Runx1 was verified in affected muscles of *mdx/Runx1* ^*f/f*^ compared to *mdx/Runx1* ^*L/L*^ mice (S2G Fig). The *mdx/Runx1* ^*f/f*^ mice represent a useful model for investigating the role of Runx1 in muscle regeneration *in vivo*. As *mdx* mice lack dystrophin expression the mice undergo recurrent cycles of muscle necrosis and regeneration. However, in contrast to human DMD patients who encounter muscle waste and paralysis at early childhood and die during the second or third decade of their lives [18], *mdx* mice exhibit extensive muscle regeneration, resulting in no loss of muscle mass, and have a normal life span (reviewed in [19]). Analysis of various litters showed that body weight of neonate and juvenile *mdx/Runx1* ^*f/f*^ are comparable to those of *mdx/Runx1* ^*L/L*^ littermates. However, starting at the age of 2 months, *mdx/Runx1* ^*f/f*^ mice did not gain weight, unlike their *mdx/Runx1* ^*L/L*^ littermates (Fig 2A). As a result *mdx/Runx1* ^*f/f*^ mice became underweighted compared to *Runx1* ^*L/L*^, *Runx1* ^*f/f*^ or *mdx/Runx1* ^*L/L*^ mice. In addition, *mdx/Runx1* ^*f/f*^ mice were notably smaller and thinner when reaching maturity (6–7 months) (Fig 2B). To evaluate the mechanism underlying this weight differences between *mdx/Runx1* ^*L/L*^ and *mdx/Runx1* ^*f/f*^ mice, we monitored their relative lean weight. Compared to *mdx/Runx1* ^*L/L*^ mice, the *mdx/Runx1* ^*f/f*^ mice have lower lean weight starting at 4 month of age and throughout the recording period (Fig 2C), suggesting that the *mdx/Runx1* ^*f/f*^ mice bear loss of muscle mass. (Fig 2C). This loss of muscle mass is consistent with the possibility that Runx1 plays a role in *mdx* related muscle regeneration.

Because loss of Runx1 seemed to affect *mdx* related muscle regeneration we assessed whether lack of muscle specific Runx1 will affect the life span of *mdx/Runx1* ^*f/f*^ mice. Kaplan-Meyer survival analysis revealed that life expectancy of *Runx1* ^*f/f*^ and *mdx/Runx1* ^*L/L*^ mice was similar to that of WT mice, whereas *mdx/Runx1* ^*f/f*^ exhibit a significantly (*p* = 4e^-291^, χ^2^ test) shorter life span with deaths occurring as early as at 12 weeks of age, with a median survival age of 28.5 weeks (Fig 2D). Histological analysis of diaphragm muscles of mice at the median survival age revealed extreme muscle deterioration, with extensive fibrosis and a pronounced decrease in diaphragm size (Fig 2E). We therefore postulate that the likely cause of death was respiratory failure. This profound reduction in life span underscores the contribution of Runx1 to the muscle pathology observed in *mdx/Runx1* ^*f/f*^ mice.

To characterize the muscular dystrophy of the *mdx/Runx1* ^*f/f*^ mice, we compared its muscle strength to that of *mdx/Runx1* ^*L/L*^ strain. It was previously reported [20] that *mdx* mice exhibits a transient decline in muscle strength at juvenile stages, which dramatically improves in mice older than 2 months. We therefore monitored muscle strength by recording treadmill performance of mice from the age of 2 to 7 months. Follow-up post-hoc comparisons (Bonferroni corrected for multiple comparisons) revealed no significant differences between WT, *Runx1* ^*f/f*^ and *mdx/Runx1* ^*L/L*^ mice at all time points. Conversely, *mdx/Runx1* ^*f/f*^ mice reached exhaustion significantly faster (*p*<0.01) than WT, *Runx1* ^*f/f*^ or *mdx/Runx1* ^*L/L*^ mice (Fig 2F). We further evaluated muscle performance by the grip strength test, which measures the maximal force a mouse can apply when gripping a rod with its forelimbs. Again, *mdx/Runx1* ^*f/f*^ mice exhibited a significant reduction (~50%, *p =* 2e^-5^, student *t-test*) in muscle strength compared to *mdx/Runx1* ^*L/L*^ mice (Fig 2G, left), regardless of their muscle mass (Fig 2G, right). These results indicate that the impaired muscle performance of *mdx/Runx1* ^*f/f*^ mice is due not only to shear loss of muscle mass, but also due to a reduction in capabilities of the remainder muscle tissue. Together, the complementary outcome of these muscle strength experiments demonstrated the importance of muscle Runx1 to *mdx* related muscle regeneration.

### Loss of Runx1 impairs muscle regeneration *in vivo*

**Fig 3:**
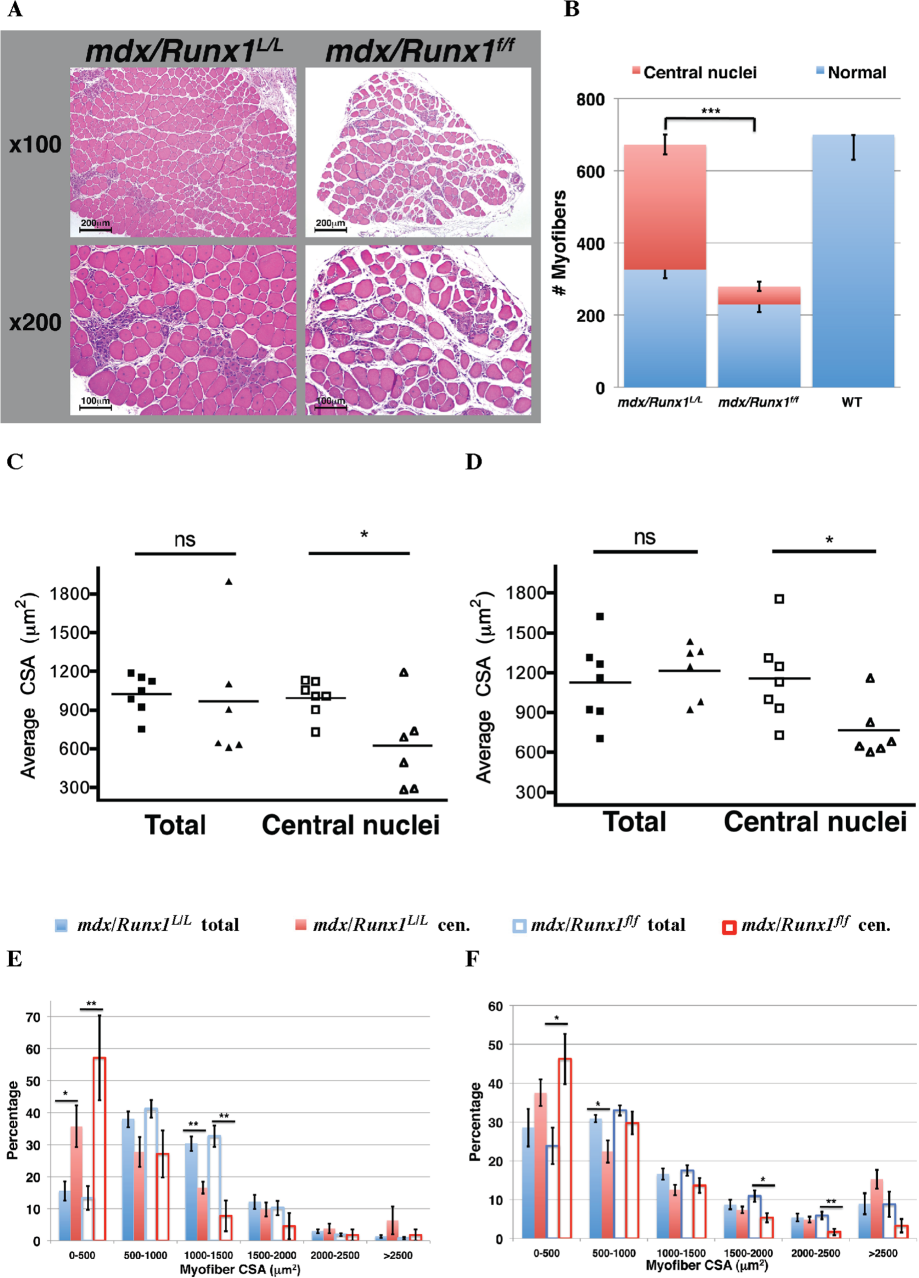
Loss of Runx1 in *mdx* mice resulted in reduced muscle regeneration. (A and B) Determination of myofibers numbers in *mdx* muscle. Soleus muscles from 8 weeks old *mdx* and *mdx/Runx1* ^*f/f*^ mice were sectioned, subjected to H&E staining, and number of total and regenerating myofibers was determined. (A) Representative images of *mdx* and *mdx/Runx1* ^*f/f*^ sections showing regenerating myofibers with central nuclei, the hallmark of regenerating myofibers, shown at x100 (top) or x200 (bottom). Scale bars, 200μm and 100μm for the x100 and x200 magnification, respectively. (B) Stacked column histograms showing the average number of regenerating myofibers (red) and normal myofibers (blue) in *mdx* and *mdx/Runx1* ^*f/f*^ soleus muscle sections. The number of regenerating (fibers with round and central nuclei) and normal myofibers was counted in 3 H&E-stained sections per muscle and their average number calculated. Values are mean±SE (n = 9–13, ***P <0.001, unpaired student *t-*test). WT myofibers number is given as negative control for the presence of regenerating myofibers. (C and D) Average CSA of total or regenerating myofibers from *mdx/Runx1* ^*L/L*^ (filled and open squares) and *mdx/Runx1* ^*f/f*^ (filled and open triangles) of 8 weeks old mice was determined for soleus (C) and gastrocnemius (D) muscles. Values are mean± SEM (*n* = 7 *mdx/Runx1* ^*L/L*^, *n* = 6 *mdx/Runx1* ^*f/f*^;*P<0.05, unpaired student *t-*test). (E and F) Quantification of CSA distribution of total or regenerating myofibers in *mdx/Runx1* ^*L/L*^ and *mdx/Runx1* ^*f/f*^: the percentage of *mdx/Runx1* ^*L/L*^ total myofibers (filled blue columns), *mdx/Runx1* ^*L/L*^ regenerating myofibers (filled red columns), *mdx/Runx1* ^*f/f*^ total myofibers (empty blue columns) and *mdx/Runx1* ^*f/f*^ regenerating myofibers (empty red columns) was determined for soleus (E) and gastrocnemius (F) muscles. Values are mean ± SEM (*n* = 7 *mdx/Runx1* ^*L/L*^, *n* = 6 *mdx/Runx1* ^*f/f*^; *, P < 0.05; **, P < 0.01, unpaired student *t*-test).

We next investigated whether the muscle wasting encountered by *mdx/Runx1* ^*f/f*^ mice involves a decrease in the number (i.e. regeneration defect) or size (i.e. enhanced atrophy/inability to produce proper hypertrophy) of myofibers. Analysis of soleus muscles of 8-week-old *mdx/Runx1* ^*f/f*^ mice revealed a significant reduction in the number of total myofibers compared to *mdx* or *mdx/Runx1* ^*L/L*^ mice (Fig 3A and 3B). Importantly, the amount of centrally nucleated myofibers, a hallmark of regenerative muscle tissue, was significantly decreased in soleus muscle of *mdx/Runx1* ^*f/f*^ mice both in terms of absolute numbers (70.2±20.58 vs. 344.38±25.36, *p* = 5.5e^-8^, unpaired student *t-test*) and percentage of total fibers (18.75±3.98% vs. 49.72±3.18%, *p* = 5.6e^-6^, unpaired student *t-test*) (Fig 3B). Interestingly, a similar reduction in centrally nucleated myofibers was also observed in CTX-treated muscle of *Runx1* ^*f/f*^ mice (S3A and S3B Fig). This data supports the possibility that loss of Runx1 leads to a decrease in muscle regeneration in *mdx/Runx1* ^*f/f*^ mice.

To evaluate whether the profound muscle waste in the *mdx/Runx1* ^*f/f*^ mice could be attributed to the ability of Runx1 to attenuate muscle atrophy, as previously observed in denervated muscle [21], we determined the total myofiber size, i.e., the average cross-sectional area (CSA), in the soleus and gastrocnemius muscles. We found no significant reduction in myofiber CSA in either muscle type of *mdx/Runx1* ^*f/f*^ mice as compared to *mdx/Runx1* ^*L/L*^ mice (Fig 3C and 3D). This finding indicates that a Runx1 function other than its role in muscle atrophy must be the underlying cause for the striking muscle waste in *mdx/Runx1* ^*f/f*^ mice. Indeed, when the regenerating myofibers were recorded separately by counting the centrally nucleated myofibers, a significant CSA reduction was noted in *mdx/Runx1* ^*f/f*^ mice muscles compared to those of *mdx/Runx1* ^*L/L*^ mice (Fig 3C and 3D). Moreover, quantitative analysis of CSA distribution revealed a significant increase of small myofibers fraction in the *mdx/Runx1* ^*f/f*^ muscles, which was more pronounced in the centrally nucleated myofiber subset (Fig 3E and 3F). The decrease in the CSA of centrally nucleated myofibers and the change in CSA distribution indicate that regenerating myofibers in *mdx/Runx1* ^*f/f*^ mice were formed by fusion of a smaller number of myoblasts, conceivably due to decreased myoblast proliferation in *mdx/Runx1* ^*f/f*^ muscles. Interestingly, a similar reduction in CSA of centrally nucleated myofibers was found in the CTX- treated muscles of *Runx1* ^*f/f*^ compared to *Runx1* ^*L/L*^ mice (S3A and S3C Fig). To directly address whether the muscle regeneration deficit of *mdx/Runx1* ^*f/f*^ and *Runx1* ^*f/f*^ mice was due to impaired myoblast proliferation, we recorded cell proliferation by BrdU staining. A significant decrease in the number of BrdU^+^ cells was observed within the damaged muscle of *mdx/Runx1* ^*f/f*^ compared to *mdx/Runx1* ^*L/L*^ muscle (S3D and S3E Fig). The reduction in proliferating myoblasts was also manifested in decreased number of Pax7^+^ cells in regenerating muscle of *mdx/Runx1* ^*f/f*^ mice compared to *mdx/Runx1* ^*L/L*^ mice (S3F and S3G Fig). We then examined SC proliferation by co-staining muscles of *mdx/Runx1* ^*L/L*^ and *mdx/Runx1* ^*f/f*^ mice with anti-Pax7 and anti-Ki67 Ab (S3H and S3I Fig). Significantly, marked reduction in the number of double positive Pax7^+^/Ki67^+^ cells was noted in muscle of *mdx/Runx1* ^*f/f*^ mice compared to *mdx/Runx1* ^*L/L*^ mice (S3I Fig, left panel). Moreover, the percentage of proliferating Pax7^+^ cells within the total SC population was also markedly reduced in muscles of *mdx/Runx1* ^*f/f*^ mice compared to *mdx/Runx1* ^*L/L*^ mice. Together, the complementary results obtained using anti- BrdU, Pax7 and Ki67 Ab demonstrate a reduced proliferation capacity of SC in regenerating muscle of *mdx/Runx1* ^*f/*^ mice compared to *mdx/Runx1* ^*L/L*^ mice. Similar phenotype was observed in CTX-treated muscles of *Runx1* ^*f/f*^ mice (S3J and S3K Fig). Collectively, these findings suggest that Runx1 promotes muscle regeneration-associated myoblast proliferation and loss of Runx1 in *mdx/Runx1* ^*f/f*^ or *Runx1* ^*f/f*^ impairs muscle regeneration causing marked muscle wasting in the *mdx/Runx1* ^*f/f*^ mice.

### Runx1 controls the proliferation/differentiation balance of primary myoblasts

**Fig 4:**
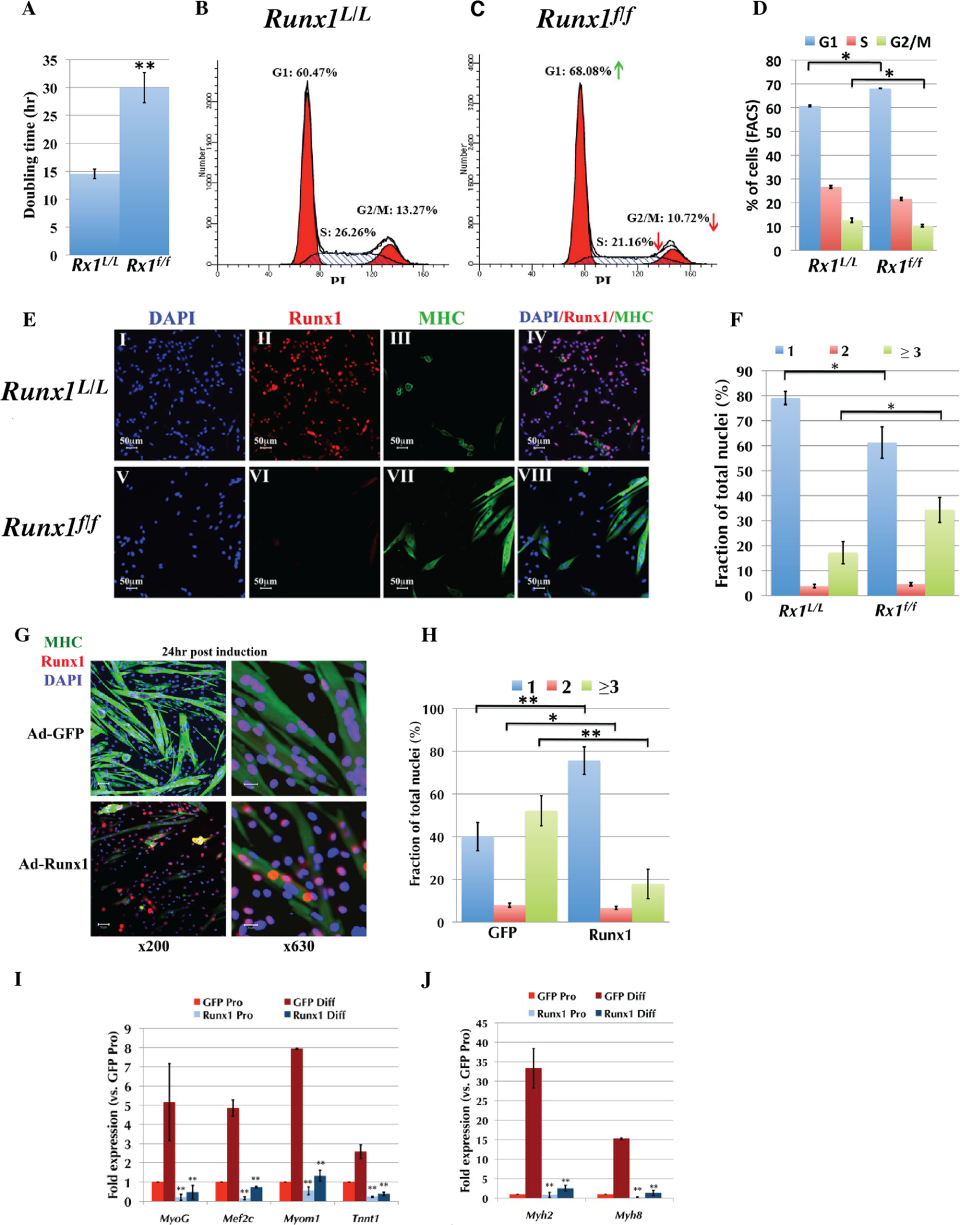
Runx1 attenuates PM proliferation. (A to F) *Runx1* ^*L/L*^ and Runx1^*f/f*^ PM were purified and their proliferation properties were compared. (A) Average doubling time of *Runx1* ^*L/L*^ and Runx1^*f/f*^ PM cultures. Values are mean±SD (n = 4, **p <0.001). (B and C) Cell cycle analysis of proliferating PM derived from *Runx1* ^*L/L*^ (B), or *Runx1* ^*f/f*^ (C) mice. Cell-cycle phases G1, S, and G2/M and the relative size (in %) of PI labeled populations out of total cells are indicated. Results from one of four *Runx1* ^*L/L*^ or *Runx1* ^*f/f*^ different cultures with similar findings are shown. Green and red arrows indicate increase in % of G1 and decrease in % of S and G2/M of *Runx1* ^*f/f*^ vs. *Runx1* ^*L/L*^ PM. (D) Histograms summarizing the distribution of cell populations as analyzed in C. Values are mean±SD (n = 4, *p <0.05). (E) IF analysis of proliferating PM from Runx1^*L/L*^ and *Runx1* ^*f/f*^ mice using anti-Runx1 and MHC Abs. (*I-IV*) *Runx1* ^*L/L*^ and (*V-VIII*) *Runx1* ^*f/f*^ at x200 magnification, scale bars, 50 μm. Results from one of four *Runx1* ^*L/L*^ or *Runx1* ^*f/f*^ different cultures with similar findings are shown. (F) Average fusion index of proliferating PM. *Runx1* ^*L/L*^ and *Runx1* ^*f/f*^ proliferating PM cultures were stained with anti-MHC Ab and DAPI and the fractions (in %) of single (blue), double (red) and multinucleated (≥ 3, green) cells are shown. Values are mean±SE (n = 4, *p <0.05). (G to J) Proliferating WT PM were infected with either Ad5CMV-eGFP or Ad-Runx1 and then grown for 24 h in differentiation medium prior to analysis. (G) IF analysis of infected PM using anti- Runx1 and MHC Abs (scale bars, 50 μm and 20 μm for x200 or x630 magnification, respectively). DAPI was used as a nuclear marker. Results from one of four different experiments with similar findings are shown. (H) Histograms showing the average fusion index of differentiating PM analyzed in (G). The fractions (in %) of single (blue), double (red) and multinucleated (≥ 3, green) cells are shown. Values are mean±SE (n = 4, **p <0.001, *p <0.05). (I and J) RT-qPCR analysis of myogenic gene expression in proliferating PM (Pro) before or 72 h post differentiation induction (Diff). PM were grown and infected as indicated in (G), RNA was purified and analyzed by TaqMan assay. Values are mean±SD (n = 3, **p <0.001).

The impaired muscle regeneration seen in both CTX-treated *Runx1* ^*f/f*^ and *mdx/Runx1* ^*f/f*^ mice is compatible with the notion that loss of Runx1 in SC-derived myoblasts leads to proliferation defects. We directly examined this possibility by culturing PM from *Runx1* ^*f/f*^ and *Runx1* ^*L/L*^ littermates under proliferation-inducing conditions. *Runx1* ^*f/f*^ PM proliferation was attenuated as indicated by the significantly longer doubling time compared to *Runx1* ^*L/L*^ PM (Fig 4A). This prolonged doubling time resulted from *Runx1* ^*f/f*^ PM arrest in the G1 phase (Fig 4B–4D). Prolonged doubling time resulted from impaired cell cycle progression was also observed in adenovirus-Cre-GFP (Adeno-Cre)-infected *Runx1* ^*L/L*^ PM (S4A–S4C Fig), underscoring the finding that lack of Runx1 is the cause for this phenotype. We then used PI staining to determine whether the cell cycle progression impairment was associated with cell death. Comparing, No reduction in *Runx1* ^*f/f*^ PM viability compared to *Runx1* ^*L/L*^ PM was noted (S4D and S4E Fig), indicating that loss of Runx1 did not induce myoblasts death.

We also evaluated the role of Runx1 in PM differentiation by analyzing the expression of myosin heavy chain (MHC), a myofiber differentiation marker using immunofluorescence (IF). Compared to *Runx1* ^*L/L*^, the *Runx1* ^*f/f*^ cultures contained a significantly higher number of MHC-positive, multinucleated myofibers (Fig 4E). This *Runx1* ^*-/-*^-dependent phenotype was further characterized by counting the number of fusion events in proliferating *Runx1* ^*L/L*^ and comparing it to that measured in *Runx1* ^*f/f*^ PM cultures (Fig 4F). *Runx1* ^*f/f*^ myoblasts displayed a significantly lower number of mononuclear cells and a two-fold increase in the amount of multinucleated myofibers (34.28±6.2% vs. 17.13±2.8%; *p* = 0.023). Similar results were obtained with cultured *Runx1* ^*L/L*^ PM infected with Ad-Cre-GFP (S4F Fig). Together, the attenuated proliferation and spontaneous differentiation of *Runx1* ^*f/f*^ PM, suggest that Runx1 participates in myoblast cell-fate decision to proliferate or differentiate and when lost the normal proliferation/differentiation balance is disturbed.

### Ectopic Runx1 expression delays myoblast differentiation

Because Runx1 affects the PM proliferation/differentiation balance, we questioned whether ectopic Runx1 expression inhibits myoblast differentiation. Cultured PM were infected with either Runx1-expressing Ad-Runx1-GFP or Ad-GFP viral constructs. IF analysis revealed fewer multinucleated MHC-expressing myotubes in the Ad-Runx1-GFP infected culture compared to those of Ad-GFP (Fig 4G and 4H), indicating that ectopic expression of Runx1 causes delayed differentiation. This result correlates with the reciprocal effect of *Runx1* ^*-/-*^, which manifested in enhanced PM differentiation (Figs 4E, 4F and S4D). To further characterize this ectopic Runx1-induced delayed differentiation phenotype, we analyzed expression of *Myog* and *Mef2c* TFs and the sarcomeric genes *Myomesin*, *Troponin T*, *Myh2* and *Myh8*, which are induced during myoblast differentiation. RT-qPCR analysis revealed reduced expression of these genes in ectopically expressing Runx1-differentiating PM (Fig 4I and 4J), supporting the observation that Runx1 expression delays myoblast differentiation.

The high expression level of Runx1 in proliferating PM (Fig 1F), prompted us to conduct a complementary assessment of its levels during PM differentiation. Western blot analysis indicated that Runx1 levels decrease during differentiation (S5A and S5B Fig) and that addition of the proteasome inhibitor Bortezomib attenuated this decline (S5C and S5D Fig). In contrast, *Runx1* RNA levels did not significantly change during myoblast differentiation, as determined by RT-qPCR (S5E Fig). We therefore conclude that Runx1 is actively degraded in differentiating myoblasts and that this breakdown facilitates myoblast differentiation.

### Identification of Runx1 target genes

**Fig 5:**
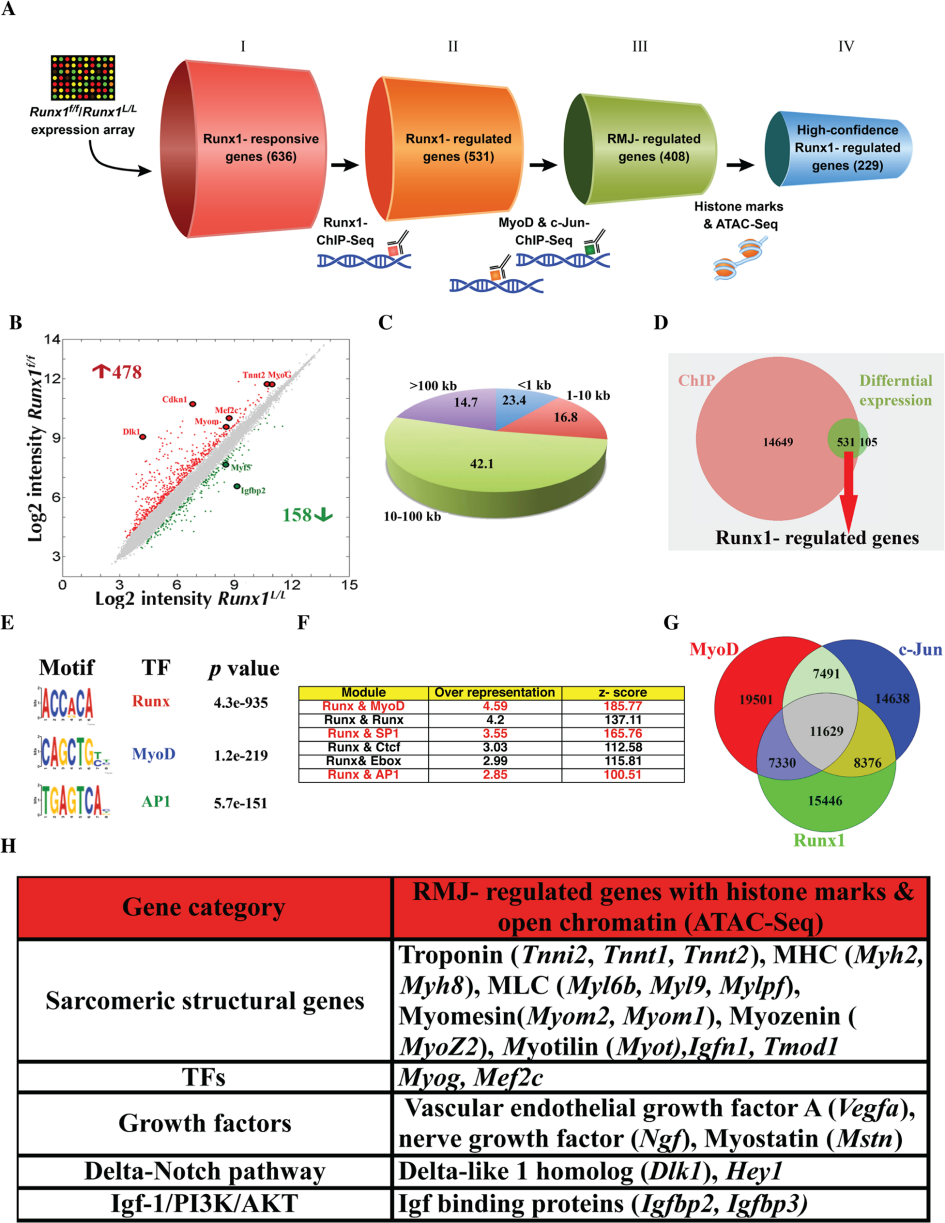
Analysis of PM high confidence Runx1-regulated genes. (A) Schematic representation of the selection procedures used to identify high-confidence Runx1-regulated genes. Each cylinder represents a gene subset, with the gene number given in brackets. I- Runx1-responsive genes were derived from *Runx1* ^*L/L*^ vs. *Runx1* ^*f/f*^ PM microarray expression data. II- Runx1-regulated genes were derived by cross analysis of the Runx1-responsive genes dataset and Runx1 ChIP-seq data. This gene subset represents Runx1-responsive genes that are also occupied by Runx1. III- RMJ-regulated genes are Runx1-responsive genes that are co-occupied by Runx1, MyoD and c-Jun. IV- High-confidence Runx1-regulated gene subset are RMJ-regulated genes that were also marked as having adjacent active regulatory elements by both anti histone modifications (H3K4me1 & H3K27ac) ChIP-seq and ATAC-seq. (B) Scatter plot of differentially expressed genes in WT vs. *Runx1* ^*f/f-*^ PM. Gene expression level (log2 scale) in *Runx1* ^*f/f*^ vs. WT PM is plotted. Significant increased or decreased genes are indicated in red or green, respectively. Filled circles indicate Runx1-responsive genes that are known to participate in myoblast proliferation or differentiation. (C) Pie chart depicting Runx1 binding sites distribution in relation to the nearest annotated TSS. Numbers represent % of bound regions. (D) Venn diagram summarizing the overlap between Runx1-ChIP-seq bound genes (ChIP) and Runx1-responsive genes, differentially expressed in *Runx1* ^*f/f*^ vs. *Runx1* ^*L/L*^. Runx1-regulated genes are defined as Runx1-bound genes that were also Runx1-responsive. (E) Enriched TF motifs among Runx1-bound regions from PM ChIP-seq data. (F) Overrepresented TF modules in Runx1-bound regions from PM. Runx1 ChIP-seq data was analyzed using the module overrepresentation tool in Genomatix package (RegionMiner). The table presents the most highly enriched modules. (G) Venn diagram showing the overlap of regions bound by Runx1, MyoD and c-Jun and the common fraction of 11629 regions. (H) Cross analysis of all ChIP seq and ATAC-seq common loci with Runx1-responsive gene list (Fig 5B). Prominent genes are presented.

We next investigated the Runx1-mediated transcriptional program involved in the early stages of muscle regeneration. A genome-wide analysis of cultured PM was perform following the strategy described in Fig 5A. First, we identified Runx1-responsive genes by comparing gene expression profiles of *Runx1* ^*L/L*^ and *Runx1* ^*f/f*^ PM. Runx1-responsive genes were defined using an FDR q-value <0.1 and >1.5-fold change as the significant threshold. The analysis revealed 636 differentially expressed genes, of which 478 were upregulated and 158 were downregulated in *Runx1* ^*f/f*^ PM (Fig 5B and S1 Table). This Runx1-responsive gene-subset contains genes known to play a role in the myoblast proliferation/differentiation balance including, the MRFs *Myf5*, *MyoG* and *Mef2c* (Fig 5B). We also noted a change in the expression levels of muscle structural genes, including Myomesin (*Myom2*) and Troponin (*Tnnt2*) isoforms that compose the sarcomere and are activated in differentiated myoblasts (Fig 5B). Other genes that were found to be differentially expressed included the signaling pathway-related genes *Dlk1* of the Delta-Notch and the *Igfbp2* of the Igf-1/PI3K/Akt pathways, and *Cdkn1c* a cell-cycle regulator encoding the cyclin-dependent kinase-inhibitor p57^Kip2^ protein (Fig 5B). Of note, in contrast to the considerable role played by Cbf-β in C2C12 myoblastic cell-line differentiation [14], no significant change was noted in *Cbf-β* levels in *Runx1* ^*f/f*^ PM (see S1 Table). These results suggest that in PM, Runx1 regulates the expression of MRFs, sarcomeric genes and cell cycle-control genes, thereby promoting myoblast proliferation and attenuating their differentiation.

Runx1-responsive gene analysis represents changes in genes that are either direct or indirect targets of Runx1. To identify genes directly regulated by Runx1 in PM we conducted Runx1 ChIP-seq using proliferating PM, which enable us to single- out Runx1-responsive genes that are bound by Runx1 (Fig 5A). Runx1 occupancy pattern displayed enrichment at promoter regions, defined as 1 kb upstream and downstream from an annotated transcription start sites (TSS). However, most Runx1-bound regions were distal to annotated TSS; 42% were located within 10–100 kb from TSS, and 15% were found in “gene deserts” (>100 kb from any TSS) (Fig 5C). All in all, most Runx1-bound regions (85% of Runx1 chip-seq peaks) were located within 100 kb from known TSS (Fig 5C) with more than 94% of the peaks located up to 200 kb from known TSS. A similar Runx1 occupancy pattern was observed in differentiating megakaryocytes [22] and in hematopoietic progenitors [23]. In C2C12 myoblasts, the median enhancer-TSS distance was defined as ~53kb [13]. We then identified the Runx1-regulated genes by cross-analyzing the Runx1-responsive gene dataset with the ChIP-Seq results. Out of the 636 Runx1-responsive genes, 83% contained Runx1-bound regions within 200kb from their TSS; these 531 genes were considered as Runx1-regulated genes (Fig 5D, S2 Table). A partial list of Runx1-regulated muscle-relevant genes is presented in S3 Table. Collectively, the gene expression and ChIP-seq analyses indicate that during myoblasts proliferation, Runx1 regulates muscle-specific genes that encode MRFs and structural proteins and that it may serve as a component of the Igf-1/PI3K/Akt and Delta-Notch pathways.

*De-novo* TF motif analysis of the Runx1-bound regions revealed a significant enrichment of the canonical RUNX motif (*p* = 4.3e^-935^) as well as MyoD and AP-1 TF motifs (Fig 5E). In fact, over 95% of Runx1-bound regions contained at least one RUNX motif. Previous studies have found cooperation between AP-1 and Runx1 in proliferating megakaryocytes [22], an enrichment between of Runx and AP-1 motifs in C2C12 cells [12] and enrichment of c-Jun and Runx1 that are recruited by MyoD to several muscle specific enhancers in C2C12 cells [13]. These findings prompted us to analyze the proliferating PM ChIP-seq data for TF module enrichment, defined as TF motifs within 50bp spanning the bound RUNX motif. Analysis revealed Runx-Runx, Runx-MyoD and Runx-AP1 to be among the most enriched modules (Fig 5F). The preponderance of the two latter modules further supports the possibility of cooperation between Runx1, MyoD and AP-1 TFs in driving the transcription program that regulates PM proliferation/differentiation balance. Analysis of Runx1-bound regions using the GREAT program [24], which predicts meaningful biological functions from the landscape of TF-bound regions, indicated enrichment for many skeletal muscle-related terms and relevant signaling pathways (S4 Table). The enrichment of MyoD and c-Jun motifs, as well as Runx-MyoD and Runx1-Ap1 modules, in Runx1 ChIP-seq data suggests that these TFs cooperate during muscle regeneration.

### Runx1, MyoD and c-Jun co-occupy genomic regions in PM

As noted above, unbiased *de novo* motif-finding analysis of Runx1-bound regions in PM revealed a significant enrichment of MyoD and AP-1 motifs as well as Runx-MyoD and Runx-Ap1 modules. To obtain a better understanding of the Runx1-mediated transcriptional program and derive the signature of Runx1 in proliferating PM we characterized the regulatory regions bound by the TFs Runx1, MyoD and c-Jun (RMJ). We started by performing independent MyoD- ChIP-seq using proliferating PM (Fig 5A). While MyoD binding was enriched at the promoter regions, it was more abundant at TSS distal regions (S6A Fig), as was also observed by Cao et al in C2C12 cells [12]. Motif-finding analysis of MyoD-bound regions revealed the MyoD motif to be the most enriched followed by the AP1 and Mef2a motifs (S6B Fig). ChIP-seq data analysis revealed a significant overlap between Runx1-occupied regions and those bound by MyoD (S6C Fig). Specifically, 46% of Runx1-bound regions were co-occupied by MyoD in PM (*p*<1e^-4^, bootstrap test) (S6C Fig). This co-occupancy of Runx1 and MyoD suggests a genome-wide cooperation of the two TFs in PM.

To further define the TF myoblast regulatory regions, we examined the genome-wide binding pattern of c-Jun in PM. Since c-Jun was implicated as a negative regulator of differentiation in the myoblastic C2C12 cell line [25,26,27] and was shown to co-bind with Runx1 and MyoD at genomic loci in these cells [13], we first examined its expression in differentiating PM. Interestingly, c-Jun mRNA and protein levels gradually decreased during myoblast differentiation (S7A and S7B Fig), reminiscent of the Runx1 decay noted before (S5 Fig). This finding corroborates the possibility that c-Jun and Runx1 cooperate during myoblast proliferation, prompting us to perform a c-Jun ChIP-seq in proliferating PM. Motif analysis revealed that c-Jun-bound regions are highly enriched for AP-1, RUNX and MyoD motifs (S7C Fig). Moreover, 47% of the c-Jun-occupied regions are co-bound by Runx1 (S7D Fig), and a substantial number of peaks were bound by all three TF (Fig 5G, *p*<1e^-4^, bootstrap test). We further characterized the Runx1-cJun co-occupied regions by conducting c-Jun ChIP-seq using PM lacking Runx1 (*Runx1* ^*f/f*^ PM). Interestingly, qPCR analysis revealed a pronounced reduction in bound c-Jun at several *Runx1* ^*f/f*^ PM loci compared to WT PM loci (S7E Fig). Of note, the observed decrease in c-Jun binding upon loss of Runx1 was not due to a reduction in c-Jun protein levels (S7F Fig). These findings support the notion that Runx1 plays a role in recruiting c-Jun to at least a portion of their co-bound sites.

### Identification of high-confidence PM Runx1-regulated genes

Cross-analysis of the RMJ-bound genomic regions with the Runx1-responsive gene subset yielded a significant (2e-^16^, hypergeometric test) list of 408 genes highly enriched for muscle-related GO terms (S5 Table), designated RMJ-regulated genes (Fig 5A). Importantly, this gene subset includes a preponderance of Runx1-repressed genes (S8A Fig), along with genes involved in myoblast proliferation and/or differentiation (S8B Fig). To further characterize the RMJ-regulated gene subset, we analyzed the epigenomic status of Runx1- and RMJ-bound regions in PM (Fig 5A). First, we performed ChIP-seq of H3K4 monomethylation (H3K4me1) and H3K27 acetylation (H3K27ac). These two histone modifications are known to mark active enhancer loci (reviewed in [28]). Analysis revealed that over 70% of Runx1-bound and 90% of RMJ-bound regions overlap with the histone marked enhancer subset (S9A and S9B Fig), underscoring the notion in PM that the three TFs occupy a subset of myoblast active enhancers that form the core of Runx1-mediated regulatory network. A more stringent analysis that enabled the identification of nucleosome-free open chromatin was achieved using the recently developed assay of ATAC-seq [29]. This evaluation showed that ~ 25% and ~40% of Runx1- and RMJ-bound regions, respectively, have a nucleosome-free structure (S9C and S9D Fig). We then cross-analyzed the combined RMJ-bound ChIP-seq data of histone-marked enhancers and ATAC-seq open chromatin regions with the 636 Runx1-responsive expressed genes (see Fig 5B). This analysis yielded a subset of 229 high-confidence Runx1-regulated genes (*p*<1e^-15^, Monte Carlo FDR) that responded to the loss of Runx1 and had RMJ-bound to adjacent nucleosome-free histone marked enhancers. This subset includes a number of major muscle regulatory and structural genes, including *Myog*, *Myh2*, *Tnnt1* and *Myom2*, the signal transduction-related genes *Dlk1*, *Hey1* (Delta/Notch pathway) and I*gfbp2*, *Igfbp3* and *prkcd* (Igf-1/AKT/mTor pathway) (Fig 5H). The finding of H3K4me1 and H3K27Ac, which mark active enhancers, at Runx1 bound loci that mediate gene silencing was puzzling. These Runx1 bound loci could represent poised enhancers that are activated upon differentiation. To test this possibility, we performed H3K27me3 and H3K4me1 qChIP that when occurred together mark poised enhancers [30,31]. While all examined loci were enriched for H3K4me1 (S9E Fig), in most of them the level of H3K27me3 in differentiated WT PM decreased (S9F Fig). Comparison of these loci in proliferating *Runx1* ^f/f^ PM to WT PM revealed similar pattern in some, but not all the loci (S9F Fig). This finding might reflect the heterogeneity of *Runx1* ^*f/f*^ PM cultures that contain both proliferating and differentiating myoblasts (See Fig 4A–4D).

To further evaluate the participation of Runx1 in regulation of the high-confidence Runx1-regulated genes we examined four RMJ-bound active enhancers regions located at the vicinity of *Myog*, *Tnnt1*, *Myh8* and *Myom2* gene loci. These four Runx1-responsive genes play key roles in muscle development and regeneration [3]. The four genomic regions were cloned into pTK-Luc reporter construct and evaluated by ectopic expression. Following co-transfection with Runx1 expression vector into HEK293 cell line, the four regions conferred Runx1-dependent repression of the basal promoter activity (S10A Fig). This Runx1-dependet repression was abrogated by mutations in the RUNX binding site of *Myog* and *Tnnt1* constructs (S10B Fig). The intact and mutated reporter constructs were also transfected into PM cultures normally expressing Runx1 (Fig 2F). In comparison to the mutated construct, activity of the intact construct was repressed (S10C Fig), presumably by the endogenous PM Runx1 binding to the intact RUNX motif.

Taken together, the histone-marked-enhancer and ATAC-seq-open-chromatin regions, the RMJ ChIP-seq and the differential gene expression results have stringently identified, at high confidence, a subset of MyoD-bound Runx1-regulated genes. These data suggest that in proliferating PM Runx1 cooperates with c-Jun to repress MyoD-activated genes that drive myoblast differentiation, and thereby participates in maintaining a proper proliferation/differentiation balance. In the absence of Runx1, this delicate equilibrium is disrupted resulting in impaired muscle regeneration.

### Validation of Runx1 target genes in *mdx* muscle

**Fig 6:**
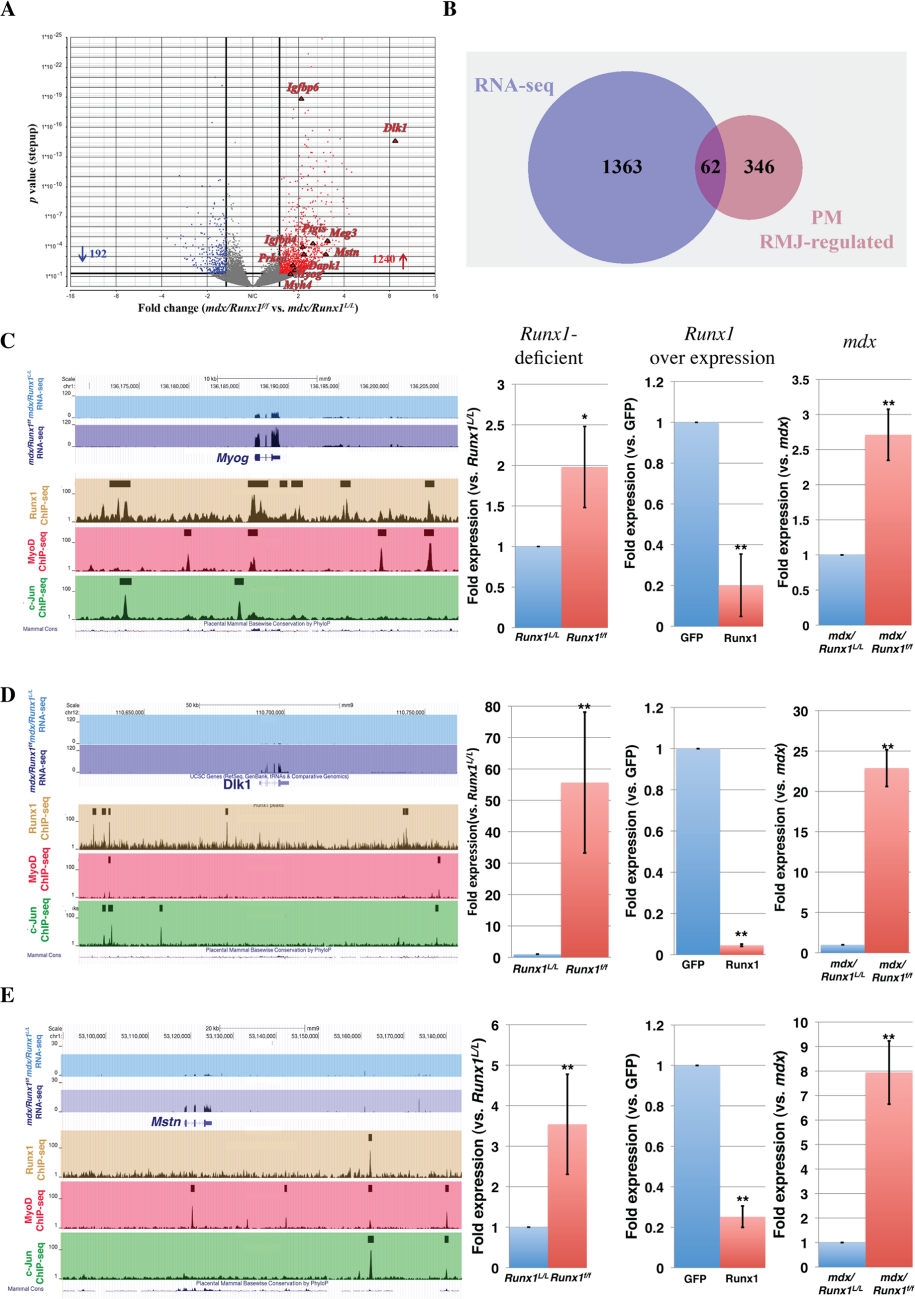
Validation of *in vivo* high confidence Runx1-regulated genes. (A) Volcano plot of differentially expressed genes in soleus muscle of 8 weeks old *mdx/Runx1* ^*f/f*^ vs. *mdx* mice. Fold expression change against *p* value is plotted. Significant increased or decreased genes are indicated in red or blue, respectively. Filled triangles indicate Runx1-responsive genes that are known to participate in myoblast proliferation or differentiation. (B) Venn diagram summarizing the overlap between *mdx* Runx1- responsive (RNA-seq) and PM RMJ- regulated gene. These genes are defined as high confidence Runx1- regulated genes in *mdx* myoblasts. (C to E) UCSC genome browser screenshots showing ChIP-Seq performed in PM and *mdx/Runx1* ^*f/f*^ vs. *mdx* mice RNA- seq tracing examples of high-confidence Runx1-regulated genes. Expression of these genes was quantified by RT-qPCR of cultured Runx1-deficient or-over expressing PM, and *in vivo* in *mdx/Runx1* ^*f/f*^ vs. *mdx* muscles. Values are mean±SD (n = 3). (C) *Myog*, encoding Myogenin (**p<0.001, *p <0.05). (D) *Dlk1*, encoding Delta-like 1 homolog (**p <0.001). (E) *Mstn*, encoding Myostatin (**p <0.001).

To gain insight into Runx1 activity in muscle regeneration *in vivo*, we analyzed the transcriptional program of Runx1 in affected *mdx* muscles. Gene expression profiles in *mdx/Runx1* ^*L/L*^ and *mdx/Runx1* ^*f/f*^ muscles were determined by RNA sequencing (RNA-seq). Runx1-responsive genes were defined using an FDR q-value <0.05 and >1.7-fold change as the significant threshold. Analysis revealed 1432 differentially expressed genes, with 1240 and 192 genes were up- and down- regulated, respectively, in *mdx/Runx1* ^*f/f*^ soleus muscles (Fig 6A and S6 Table). Among the *mdx* Runx1-responsive genes were muscle-related genes such as *Myog*, sarcomeric structural genes (*Myh7*, *Myh4*, *Myh3 Mybph*, *Tnnt2*) and signal transduction pathway-related genes such as *Igf1*, *Igfbp4*, *Igfbp6* (Igf-1/PI3K/Akt pathway) *Dlk1* (Delta- Notch pathway) and *Mstn* (Myostatin- of the Tgf β family). This list corresponds with the Runx1-responsive gene subsets found in PM.

To identify genes directly regulated by Runx1 in DMD-induced muscle regeneration, we cross-analyzed the *mdx/Runx1* ^*f/f*^ gene expression data with the *Runx1* ^*f/f*^ PM RMJ-regulated gene subset. This analysis yielded 62 genes (Fig 6B and S7 Table), representing a high-confidence Runx1-regulated gene subset in *mdx/Runx1* ^*f/f*^ soleus muscle. Fig 6C–6E depicts examples of ChIP-seq and RNA-seq readouts of three high-confidence Runx1-regulated genes *Myog*, *Dlk1* and *Mstn* all known to participate in myoblast proliferation/differentiation balance. The expression of these genes was verified by RT-qPCR in *Runx1* ^*f/f*^ PM, *mdx* muscles and PM overexpressing Runx1 (Fig 6C–6E right panels). In summary, using differential gene expression acquired in the *mdx* mice combined with the PM-derived expression and ChIP-seq data we were able to identify a subset of high-confidence Runx1-regulated genes participating in myoblast proliferation/differentiation balance. In *mdx* mice lacking Runx1, normal regeneration is impaired, leading to the adverse muscle waste phenotype of *mdx/Runx1* ^*f/f*^ mice.

## Discussion

Muscle regeneration following injury is mediated by the activation, proliferation and differentiation of adult SCs [2]. Maintaining the balance between myoblast proliferation and differentiation is crucial for proper muscle regeneration heighted by the fact that insufficient proliferation causes a reduction in myoblast pool leading to incomplete reconstitution of muscle mass. Indeed, disruption of the proliferation/differentiation equilibrium results in an impaired regeneration phenotype characteristic of muscle-wasting diseases [32,33,34].

**Fig 7:**
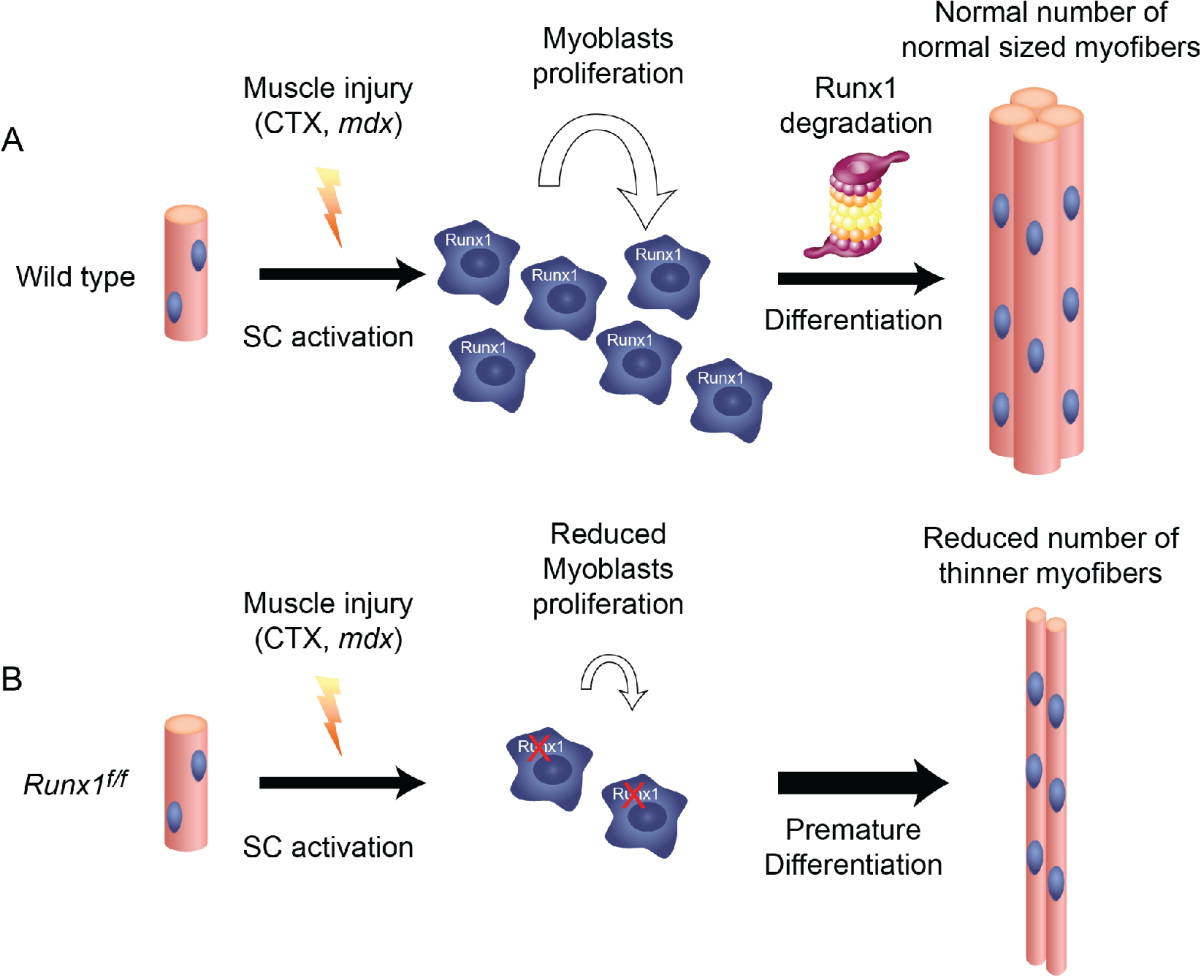
Runx1 is required for myoblast proliferation during muscle regeneration. Schematic diagram summarizing the scenario of Runx1-regulated myoblast proliferation during muscle regeneration: (A) Following myonecrosis of WT muscle, SC are activated, Runx1 is induced and promote proliferation and prevents premature differentiation. Once the critical mass of myoblasts is reached Runx1 is destined to degradation, myoblasts differentiate to produce normal size myofibers. (B) In *Runx* ^*f/f*^ muscles, myoblasts lack Runx1 expression and therefore undergo premature differentiation. This leads to insufficient myoblast pool size, resulting in reduced number and size of myofibers and impaired muscle regeneration.

In this study we perform an in-depth investigation of the function of Runx1 in muscle regeneration and its role in regulating myoblast proliferation/differentiation balance. While Runx1 is not expressed in normal healthy muscle, its expression is highly induced by different types of muscle damage. Muscle-specific ablation of *Runx1* in *mdx/Runx1* ^*f/f*^ mice impairs muscle regeneration *in vivo*. This diminished regeneration causes a decrease in the number and size of regenerating myofibers, leading to loss of muscle mass and motor capabilities. Similarly, CTX-induced muscle damage in muscle-specific Runx1-deficient *Runx1* ^*f/f*^ mice results in decreased myoblast proliferation relative to *Runx1* ^*L/L*^ mice. Consequently, the number of regenerative fibers and their size in CTX-treated *Runx1* ^*f/f*^ mice are reduced compared to *Runx1* ^*L/L*^ mice. At the cellular level, loss of Runx1 delays PM proliferation by affecting their cell cycle: *Runx1* ^*f/f*^ PM linger in the G_1_ phase and consequently, spontaneously differentiate. Interestingly, differentiation of WT PM is associated with gradual Runx1 degradation, suggesting that Runx1 tapering plays a role in the progression of myoblast regeneration. The finding that forced expression of Runx1 reduces PM capacity to differentiate, supports this notion. These results indicate that Runx1 prevents premature differentiation of proliferating myoblasts, thereby facilitating the buildup of the myoblast pool required for proper regeneration. Upon induction of differentiation, Runx1 is degraded allowing myoblasts to differentiate (Fig 7).

Having shown the pivotal role of Runx1 in regulating the balance of myoblast proliferation/differentiation, we used cultured PM to derive a Runx1 genome-wide occupancy pattern and identify its regulated genes during the early stages of muscle regeneration. Sequence analysis of Runx1-occupied regions revealed enrichment for the RUNX, MyoD and AP-1/c-Jun motifs. This finding corresponds with the observation that in the C2C12 myoblastic cell line, Runx1, MyoD and c-Jun co-bind to the same genomic loci [13] and supports the possibility that in PM the three TFs cooperate to prevent premature myoblast differentiation. ChIP-seq of Runx1-, MyoD- and c-Jun-occupied regions revealed Runx1-responsive genes bound by RMJ to be highly enriched for genes involved in myogenesis. These findings underscore the notion that Runx1 cooperates with MyoD and c-Jun to attenuate myoblast differentiation. Specifically, it suggests that during early regeneration RMJ cooperate to activate PM proliferation genes and repress genes that drive myoblast differentiation, thereby affecting the proliferation/differentiation balance. This could occur through repression by Runx1 and c-Jun of MyoD pro-differentiation target genes. Following Runx1 and c-Jun degradation, repression is relieved, allowing MyoD-mediated differentiation to proceed [12].

We then derived differential gene expression of *mdx/Runx1* ^*L/L*^ and *mdx/Runx1* ^*f/f*^ muscles and cross-analyzed this data with the RMJ-regulated gene subset of *Runx1* ^*f/f*^ PM. This analysis singled out a small subset of 62 genes, which we defined as *in vivo* high-confidence Runx1-regulated genes. This subset included several groups of genes known to affect myoblast proliferation/ differentiation balance providing clues regarding the mechanisms underlying the function of Runx1 in muscle regeneration. For example the muscle-related TFs, *Myog* and *Mustn1*. *Myog* (encoding Myogenin) is a myoblast differentiation-promoting MRF [3]. Thus, its repression by Runx1 would prevent premature myoblast differentiation. *Mustn1* (Mustang, Musculoskeletal Embryonic Nuclear Protein) encodes a nuclear protein highly expressed in adult regenerating muscle [35]; its knockdown in C2C12 cells inhibits cell differentiation [36]. Therefore, *Mustn1* repression by Runx1 would again prevent myoblasts differentiation. Another interesting group of high-confidence *in vivo* Runx1-regulated genes is the signal transduction pathways-related genes, including members of the Delta-Notch, Igf/Akt/mTor and Tgf-β pathways. The Delta/Notch pathway is activated by the Delta like 1 (*Dll1*), which upon binding to the Notch receptor, induces an anti-differentiation signal by upregulating *Hey1* and *MyoD*, which in turn, prevent the expression of pro-myogenic genes [37]. Interestingly, in *Runx1* PM and *mdx* muscles, we found that Delta- like homolog 1 (*Dlk1*), a putative Delta- Notch antagonist [38], to be significantly upregulated. As it was previously shown that *Dlk1* inhibits proliferation in avian [39] and mouse [40,41] myoblasts it is tempting to speculate that Runx1 participates in the myoblast Delta-Notch signaling pathway by repressing the antagonist *Dlk1* thereby promoting Delta-mediated myoblast proliferation. In case of the Igf-1/Akt/mTor pathway, which regulates muscle hypertrophy [42] and SC proliferation and differentiation [43], we found the two isoforms of Igf-1 downstream mediator protein kinase C (PKCβ and PKCδ) among the *in vivo* high-confidence gene subset. PKCδ (*Prkcd*) specific inhibition delays differentiation of C2C12 cells and primary human myoblasts [44]. In the rat myoblastic cell line H9c2, PKCδ is activated during differentiation, and its knockdown results in reduced myoblast differentiation [45]. Runx1 could regulate the pro-differentiation branch of the Igf-1 signal by repressing PKC isoforms, especially PKCδ. Finally, we found that Myostatin (*Mstn*, *Gdf-8*), a member of the Tgf-β family, is repressed by Runx1. Expressed in muscle *Mstn* serves as a negative regulator of muscle mass [46] and as attenuator of myoblast [47] and SC proliferation [48]. Ablation of *Mstn* improves muscle regeneration [49], and has been proved beneficial in *mdx* mice [50]. Therefore, Runx1 repression of *Mstn* in dystrophy-induced muscle regeneration could promote myoblasts proliferation.

The data we obtained from both *in vivo* and *in vitro* systems show Runx1 function during muscle regeneration to promote myoblast proliferation by repressing myoblast differentiation-inducing genes. Its activity in regenerating muscle is therefore required for the production of the critical amount of myoblasts needed for proper restoration of muscle mass (Fig 7A). Runx1 expression is confined to the PM proliferation stage, which mimics the first stages of muscle regeneration. Thus, it is conceivable that its affect is manifested during the first days post myonecrosis. Loss of Runx1 activity leads to premature myoblast differentiation, resulting in the diminution of the myoblasts pool and subsequent impaired regeneration (Fig 7B). Of potential relationship, Runx1 promotes the proliferation of adult stem cells in other tissues. For example Runx1 promotes adult hair follicle stem cell proliferation thereby increase the cell pool size prior to terminal differentiation [51]. In mesenchymal stem cells, RUNX1 is induced upon activation by an TGF-β signal and drives progenitor cells proliferation and restricts terminal differentiation into myofibroblast [52].

Prior work addressing Runx1 function using the C2C12 cell line or rhabdomyosaracoma myoblasts resulted in conflicting conclusions. While the C2C12 cell data [14] supported an anti- differentiation function of Runx1 the human rhabdomyosarcoma cell data [15,53] indicated that Runx1 promotes myoblast differentiation and that Runx1, MyoD and c-Jun cooperate to induce this differentiation [13,54]. However, in the PM cultures describe here the protein level of both Runx1 and c-Jun decreased at the onset of differentiation, rendering a potential role in later stages of differentiation unlikely. Additionally, c-Jun [26] and another AP-1 family member, Fra-2 [25] were found to repress myoblast differentiation. All in all, this discrepancy may have resulted from intrinsic differences between *in vivo* mouse models and cultured PM stem cells, and transformed/ immortalized myoblastic cell lines.

In summary, our findings support the conclusion that in response to injury, muscle Runx1 is switched on and cooperates with MyoD and c-Jun in order to regulate a muscle regeneration transcription program that involves changing the proliferation/differentiation balance by repressing genes that participate in myoblast differentiation. These data add unique insights into the transcriptional program driving muscle regeneration and implicate Runx1 as a potential participant in the pathology of muscle wasting diseases.

## Materials and Methods

### Ethics statement

The experiments were conducted in strict accordance with the recommendations of the US National Institutes of Health Guide for the Care and Use of Laboratory Animals. The protocols were approved by the Committee on the Ethics of Animal Experiments of the Weizmann Institute of Science (Permit Number: 01190113–2, 12720814–3). All surgery was performed under Ketamine/Xylazine anesthesia, and all efforts were made to minimize suffering.

### Mice

*Runx1* ^*f/f*^ mice were generated by crossing *Myf5*::*Cre* mice [17] onto *Runx1* ^*L/L*^ C57bl/6 mice [16,55]. *mdx/Runx1* ^*f/f*^ mice were generated by crossing *Runx1* ^*f/f*^ mice onto *mdx* mice [56]. Transgenic SOD1 mutant mice (B6.Cg-Tg (SOD1*G93A)^dl^ were obtained from Jackson Laboratory, USA and bred on C57Bl/6. Genotypes were determined by PCR of tail tissue. Mice weight was monitored once a month. Kaplan- Meyer survival curve was calculated using the PRISM© software. For body composition measurements, we used EcoMRI- 100H analyzer (Echo medical systems, USA). Mice body composition was measured monthly, as an average of three separate measurements for each mouse. For muscle damage experiments, 0.75 μg CTX (Sigma-Aldrich, Israel) in 50 μl of sterile phosphate-buffered saline was injected into the right gastrocnemius muscle of 8 weeks old mice. To record cell proliferation *in vivo*, 10 mg/ml BrdU (150 μl/30 g mouse) was injected intraperitoneally, gastrocnemius muscles were harvested 24h (in *mdx* mice) or 2h (in CTX treated mice) post BrdU injection and subjected to BrdU IHC using anti BrdU antibody (Ab) (#MCA2060, Serotec, UK).

### Satellite cell and primary myoblast cultures

PM cultures were established as previously described [57], following isolation of SC from gastrocnemius muscles of 2–3 week old C57bl/6 *Runx1* ^*L/L*^ or *Runx1* ^*f/f*^ mice. Muscles were treated with collagenase type I (C-0130, Sigma-Aldrich, Israel) for 3h and isolated myofibers were then seeded in proliferation media (BIOAMF-2, Biological Industries, Israel), in GHR Matrigel (BD Biosciences, USA) coated plates for 3 days, to facilitate SC delamination. PM were enriched by three stages of pre-plating. Cells were grown at 37°C, 5% CO_2_ on GHR Matrigel in proliferation medium, which was replaced daily. For differentiation assay, cells were grown as above to 75–80% confluency and then induced to differentiate by serum starvation in differentiation media (DM): DMEM containing 4% horse serum (Gibco, UK) and 0.04U/mL human Insulin. DM was replaced after 48h when needed. For analysis of immunostained myoblasts, 2x10^4^ cells were seeded and grown for 16 h on a Lab-Tek 8-well chamber slide, pretreated with Matrigel. For viral infection, cells were exposed for 24 hours to 6.5×10^7^ virus particles/ml of either Ad5CMV-eGFP, Ad5CMVCre-eGFP (both from Gene Transfer Vector Core, University of Iowa USA) or Adeno-Runx1 constructed in house using the AdEasy system [58]. For determination of average cell doubling time, 10^5^ primary SC/sample were plated, grown for 48h in proliferation medium and counted at the end point. For cell cycle analysis, SCs were grown under proliferation conditions for 48h, until reaching 70% confluency. Myoblasts were then fixed using cold ethanol, stained with propidium iodide (PI), and analyzed by FACS. For measurement of cell death, PM cultures were collected and washed twice with PBS and FACS analysis was performed promptly following addition of PI. As positive control of PI staining, WT PM were permeablized by incubation at 65°C for 2 min followed by mixing with untreated PM at a 1:3 ratio.

### Immunohistochemistry and immunofluorescence analysis

IHC of mouse tissue paraffin sections and of satellite cell cultures were performed as previously described [57]. Primary antibodies (Abs) used included mouse anti-MHC (MH-20, Developmental Studies Hybridoma Bank, USA at 1:5 dilution), mouse anti-Pax7 (Developmental Studies Hybridoma Bank, USA at 1:1000 dilution), rabbit anti-MyoD (sc-304, Santa Cruz Biotechnology, USA at 1:100 dilution), rat anti BrdU (MCA2060, Serotec, USA, 1:100), rabbit anti-Ki67 (275R, Cell Marque, 1:200) and our in-house affinity purified rabbit anti-Runx1 (at 1:100 dilution) [5]. HRP based IHC was performed using MOM kit (PK-2200, Vector, USA) according to the manufacturer’s instructions. Immunofluorescence analysis was performed using Cy2-, Cy3-, or Cy5-conjugated secondary Abs (Jackson ImmunoResearch, USA), at a dilution of 1:200–1:500. Images were acquired using a Zeiss LSM510 confocal microscope. For recording BrdU^+^ cells or regenerating myofibers, we subjected relevant sections to either anti BrdU IHC or H&E staining, respectively. The stained sections were photographed using a Nikon E800 light microscope, coded and manually counted by an unbiased estimator. For determining the average size of myofibers, sections were stained with H&E and the CSA of 400–500 fibers was measured by an unbiased estimator using the “count” procedure of ImagePro^+^ software. For recording Pax7^+^/Ki67^+^ cells, sections were reacted with anti-Pax7 and anti-Ki67 Ab and analyzed using the Zeiss LSM780 confocal microscope. Number of Pax7^+^, Ki67^+^ and Pax7^+^/Ki67^+^ cells was determined using the Fiji software (ImageJ 1.47v, NIH, USA). For fusion index-determination assay, 2x10^4^ primary myoblasts were transferred to chamber slides and grown in either Bio-AMF2 or DM, as indicated. Cultures were coded and stained for MHC and DAPI. Single, double and multinucleated cells were counted by an unbiased estimator using 4 biological repeats per experiment, comprising 12 different fields per repeat.

### Muscle strength and performance

Treadmill assay was performed by monthly training on a treadmill (Panlab Mouse 5-Lane Treadmill; model#: 760309; HARVARD APPARATUS, USA) over a period of 8 months, starting at the age of 2 months. Mice ran on the treadmill at 20 degrees uphill, starting at a speed of 10 meters/min. After 10 minutes, the speed was increased gradually to a final speed of 20 meters/min. The mice then ran for an additional 10 minutes at this speed. Performance was determined by comparing running time till exhaustion (defined as stepping off the running lane 5 times with less than 0.5 sec. intervals). Performance of each mouse was recorded at three consecutive days. Differences in treadmill performance at the ages of 2–9 months were assessed by one factor ANOVA (analysis of variance) for Gene (the four genotype groups) in each time point. The analyses were performed using IBM^®^ SPSS^®^ Statistics version 20.0. For grip strength assay, we use the TSE grip strength meter (#303500, TSE systems, Germany). 4 months old mice grip strength was monitored at three consecutive days, 5 times each day (15 measurements per mouse).

### Real time qPCR

cDNA was synthesized by superscript II RT kit (#18064–022, Invitrogen, USA) using 1μg of purified RNA and analyzed by qPCR using light cycler 480 (Roche, US). The following Taqman gene expression assays (Applied Biosystems, USA) were used to quantify RNA level: Mm01213404_m1 and Mm0123405_for *Runx1*, Mm0044614_m1 for *Myog*, Mm01340842_m1 for *Mef2c*, Mm00500665_m1 for *Myom2*, Mm00449089_m1 for *Tnnt1*, Mm01332564_m1 for *Myh2*, Mm01329494_m1 for *Myh8* and Mm00446973_m1 for *Tbp1*, used as an internal calibrator. Other genes were quantified using miScript SYBR green PCR kit (#218073, Qiagen, Germany). The primers used are detailed in S5 Table. Each qPCR experiment consisted of three biological repeats each using two cDNAs independently prepared. Statistics were performed using the Excel based REST software.

### Western blotting

Nuclear protein extracts were obtained following collection and sonication of cultured SCs as previously described [59]. WB was performed using our in house anti-Runx1 (1:5000) as described [5]. Primary Abs used included rabbit anti- c-Jun (sc-1694, 1:1000), rabbit anti-Emerin (sc-15378, 1:10^4^) (Santa Cruz Biotechnology, USA) and mouse anti-GAPDH (MAB374, Chemicon, USA, 1:1000). Secondary Abs used were either anti-rabbit HRP or anti-mouse HRP (Jackson ImmunoResearch, USA). Quantification of WB protein bands was conducted using the Image Quant LAS4000 (GE) device and endogenous Image Quant TL software.

### Transcriptome data acquisition and analysis

RNA was isolated by PerfectPure RNA tissue kit (# 2302410, 5 PRIME, Germany) according to the manufacturer's instructions. Purified RNA was reverse-transcribed, amplified, and labeled with Affymetrix GeneChip whole transcript sense target labeling kit. Labeled cDNA was analyzed using Affymetrix Mouse Gene 1.0 ST microarrays, according to the manufacturer's instructions. Microarray data were analyzed using Partek Genomic Suite software. CEL files (containing raw expression measurements) were imported and data was preprocessed and normalized using the Robust Multichip Average (RMA) algorithm [60]. To identify differentially expressed genes ANOVA was applied and genes fold-changes were calculated.

For RNA-seq analysis RNA was isolated from 2 months old mice Soleus muscle extracts using the PerfectPure RNA tissue kit, as mentioned above. Illumina TruSeq® RNA Sample Preparation v2 was used according to manufacturer's instructions. Indexed samples were sequenced in a Illumina HiSeq 2500 machine in a single read mode. The obtained reads, 50 bp long, were mapped to the mm9 mouse genome assembly using TopHat2 [61]. Version 2.0.12.0.10 with default options. Expression at the gene level was quantified by applying HTSeq (version 0.6.1) [62], and using the known genes from UCSC in gtf format as annotation. Differential expression was calculated utilizing the DESeq2 software (version 1.2.10) [63].

### ChIP-seq

ChIP was performed essentially as described [22]. Briefly, cross-linked chromatin from approximately 1.2x10^8^ freshly isolated primary WT myoblasts was prepared and fragmented to an average size of approximately 200 bp by 35 cycles of sonication (30 seconds each) in 15-ml tubes using the Bioruptor UCD-200 sonicator (Diagenode, USA). The following Abs were used for immunoprecipitation of fragmented chromatin: 170μl of in house anti-Runx1; 24μg of mouse anti-MyoD (sc-32758, Santa Cruz Biotechnology, USA); 24μg of rabbit anti c-Jun (sc-1694, Santa Cruz Biotechnology, USA). Rabbit pre-immune serum or mouse IgG (278–010, Ancell), were used as control for Runx1 or MyoD and c-Jun ChIP-seq, respectively. DNA was purified using QIAquick spin columns (QIAGEN) and sequencing performed using Illumina HiSeq 2500. Two biological repeats were conducted and separately sequenced for each ChIP-seq experiment. For ChIP-seq analysis, the reads were aligned to the mouse genome (mm9) allowing one mismatch and using the Bowtie aligner [64]. Reads with a unique best alignment were retained for further processing. Immunoprecipitated samples were compared against the negative control to find binding sites using the MACS software with default parameters [65].

### qChIP

ChIP products of *Runx1* ^*L/L*^ and *Runx1* ^*f/f*^ PM were purified as described for ChIP-seq using 3x10^7^ freshly isolated PM per reaction. ChIP products and input DNA were diluted to the same DNA concentration and subjected to qPCR using SYBR-green (miScript #218073, Qiagen, Germany). Each experiment consisted of three biological repeats, and input DNA served as control. Statistics were performed using the Excel based REST software.

### ATAC-seq

ATAC was performed as previously described [29]. Briefly, 5x10^4^ freshly isolated PM were harvested, and underwent the recommended transposition protocol without the lysis stage. The resulting transposed DNA was enhanced using 12 cycles of PCR, as described. The resulting libraries were sequenced using Illumina HiSeq 2500. For ATAC-seq analysis, we used similar parameters as for the ChIP-seq (see above).

### Reporter assay

For dual Luciferase assay, Runx1 bound genomic DNA fragments related to the *Myog*, *Tnnt1*, *Myh8* and *Myom2* genes were generated by PCR using primers listed in S8 Table. RUNX binding site in the *Myog* and *Tnnt1* regulatory elements was mutated by overlap PCR using primers indicated in S8 Table. Intact and mutated genomic elements were cloned into the Renilla Luciferase expression vector pTK-Luc, upstream to the TK promoter, using HindIII and BamHI restriction sites. HEK293 cells in 24-well plates were co-transfected using Lipofectamine 2000 according to the manufacturer protocol (#11668–027, Invitrogen) with 1μg of the reporter vector, 1μg of expression vector (empty pcDNA3.1 or pcDNA3.1-Runx1) and 0.01μg of pGL4.13 vector carrying firefly Luciferase as internal transfection control. PM were co-transfected in 24- well plates with 1μg of the reporter vector and 0.01μg of pGL4.13, using the Nepa21 electroporation system (Nepagene) at the following settings: Poring phase of 2 pulses of 225V for 2.5ms with a 50ms interval, followed by a transfer phase of 5 pulses of 30V for 50ms with a 50ms interval. Firefly and Renilla Luciferase activities were measured 24 h after transfection using a dual luciferase assay kit (Promega).

### Bioinformatic analysis

Ingenuity Pathway Analysis tool (https://apps.ingenuity.com/) was used for GO annotation of Runx1-regulated genes and GREAT software [24] was used for Chip-seq peak GO analysis. MEME-ChIP suit (http://meme.nbcr.net/meme4_6_1/cgi-bin/meme-chip.cgi/), was used for *de-novo* motif finding in ChIP-seq TF-bound regions with default parameters and Genomatix Genome Analyzer RegionMiner tool (http://www.genomatix.de/solutions/genomatix-genome-analyzer.html) was used for deriving overrepresented TF modules. All microarray, ChIP-seq and ATAC-seq data are available in the GEO public database under the SuperSeries accession number GSE56131.

## Supporting Information

S1 Fig Expression of Runx1 in developing muscle and cultured PM. Runx1 expression was analyzed using co-IF of Runx1 with muscle specific markers. (A) Transverse paraffin sections of E10.5 WT embryo stained using anti- MyoD and anti- Runx1 Abs. DAPI staining was used as a nuclear marker. IF analysis of limb bud muscle (left panels) and fetal liver (right panels) is shown. Shown images are at X200. Scale bars, 50 μm. (B) IF analysis of cultured proliferating PM using anti- Runx1 and Pax7 Abs. DAPI staining was used as a nuclear marker. Shown images are at X200, scale bars, 50 μm.
(TIF)

S2 Fig Muscle-specific inactivation of Runx1 in *Runx1* ^*f/f*^ */Myf5*::*Cre* mouse strains. (A) *Runx1* ^*f/f*^ and *mdx/Runx1* ^*f/f*^ breeding strategy. Left panel: muscle specific Runx1-defient mice were generated by crossing *Runx1* ^*Lox/Lox*^ (*Runx1* ^*L/L*^) mice onto *Myf5*::*Cre* mice, resulting in *Runx1* ^*f/f*^ */Myf5*::*Cre* (*Runx1* ^*f/f*^); Right panel: Runx1 and Dystrophin double KO mice were generated by crossing *Runx1* ^*f/f*^ mice onto *mdx* mice, resulting in *mdx/Runx1* ^*f/f*^ mice. (B-D) Muscle specific ablation of Runx1 was examined by measuring RNA and protein levels in muscle and thymus of *Runx1* ^*f/f*^ mice compared to WT *Runx1* ^*L/L*^ mice. (B and C) RT-qPCR analysis using a TaqMan primer that spans *Runx1* fourth exon, which is absent in *Runx1* ^*f/f*^ mice. (B) *Runx1* ^*L/L*^ and *Runx1* ^*f/f*^ mice were denervated by sciatic nerve transection. The right sciatic nerve was exposed and transected leading to denervation of the entire right hind limb. Gastrocnemius muscles were harvested from untreated and denervated muscles of *Runx1* ^*L/L*^ mice and *Runx1* ^*f/f*^ mice (14 days post treatment) and RNA was purified and quantified by RT-qPCR. A profound upregulation of *Runx1* RNA is observed in denervated *Runx1* ^*L/L*^ but not in *Runx1* ^*f/f*^ muscle (n = 3, ±SD), **P<0.001). (C) RT-qPCR of RNA purified from *Runx1* ^*L/L*^ and *Runx1* ^*f/f*^ thymi showing comparable levels of *Runx1* in both genotypes (n = 5, ±SD). (D) Images showing Runx1 IHC of thymus and denervated (Den) gastrocnemius muscle from *Runx1* ^*L/L*^ and *Runx1* ^*f/f*^ mice. Runx1 positive cells are visualized by brown nuclear staining. While both genotypes show positive Runx1 expression in the thymus, only *Runx1* ^*L/L*^ but not *Runx1* ^*f/f*^ mice show positive staining in denervated muscle sections. Results from one of four *Runx1* ^*L/L*^ or *Runx1* ^*f/f*^ mice analyzed with similar findings are shown. (E) Total body weight of postnatal *Runx1* ^*L/L*^ and *Runx1* ^*f/f*^ up to an age of 9 months. Values are mean±SD (n = 12). (F) Average myofiber size as cross sectional area (CSA) of myofibers from untreated *Runx1* ^*L/L*^ and *Runx1* ^*f/f*^ mice. Values are mean±SE (n = 7). (G) Images showing Runx1 IHC in diaphragm muscle sections of *mdx* (*mdx/Runx1* ^*L/L*^) and *mdx/Runx1* ^*f/f*^ using same conditions as described in (D).
(TIF)

S3 Fig Analysis of Runx1 function in CTX- and mdx- induced muscle regeneration. (A to C) Determination of muscle regeneration in CTX treated muscle. *Runx1* ^*L/L*^ and *Runx1* ^*f/f*^ */Myf5*::*Cre* mice were treated with CTX and 14 days later gastrocnemius muscles were sectioned and stained with H&E. (A) Images of *Runx1* ^*L/L*^ and *Runx1* ^*f/f*^ */Myf5*::*Cre* sections showing regenerating myofibers (marked by black arrows) with central myonuclei (marked by yellow asterisks). Scale bars, 50 μm. Results from one of seven *Runx1* ^*L/L*^ or *Runx1* ^*f/f*^ mice analyzed with similar findings are shown. (B) Histograms showing the average number of regenerating myofibers/section. The number of regenerating myofibers (fibers with round and central nuclei) was counted in five H&E-stained sections per treated muscle and their average number per section was calculated. Values are mean±SE (n = 7 mice of each strain, *p <0.05). (C) Average CSA of regenerating myofibers from CTX treated *Runx1* ^*L/L*^ and *Runx1* ^*f/f*^ */Myf5*::*Cre* mice was determined. Values are mean±SE (n = 7, *p <0.05). (D and E) Analysis of cell proliferation in *mdx* mice. Eight weeks old *mdx* and *mdx/Runx1* ^*f/f*^ mice were analyzed for BrdU incorporation. (D) Representative images of *mdx* and *mdx/Runx1* ^*f/f*^ gastrocnemius muscle sections stained with anti-BrdU Ab. Scale bars, 100 μm. (E) Histograms showing the average number of BrdU^+^ cells/section. BrdU^+^ cells were counted in several sections spanning 1 mm length of *mdx* or *mdx/Runx1* ^*f/f*^ muscle. Values are mean±SEM (n = 6–10, **P <0.01, unpaired student *t-*test). (F and G) Analysis of Pax7^+^ SC in muscle of *mdx* mice. Diaphragm muscles of two months old mice were subjected to IHC using anti-Pax7 Ab and the number of Pax7^+^ cells was recorded. (F) Images of *mdx* and *mdx/Runx1* ^*f/f*^ diaphragm muscle sections stained with anti-Pax7 Ab. Scale bars, 100 μm. (G) Histograms showing the average number of Pax7^+^ cells/section, as in (E). Values are mean±SEM (n = 6–7, *P <0.05, unpaired student *t-*test). (H and I) Analysis of proliferating SC in *mdx* mice. Diaphragm muscle of two months old mice were subjected to co-IF using anti-Pax7 and anti-Ki67 (proliferation marker) Ab. Numbers of Pax7^+^ and Pax7^+^/Ki67^+^ positive cells were recorded. (H) Images of *mdx* and *mdx/Runx1* ^*f/f*^ diaphragm muscle sections co-stained with anti-Pax7 and anti-Ki67 Ab. DAPI staining was used as a nuclear marker. Shown are images at X400, scale bars 50 μm. (I) Left panel: Histogram showing the average of double positive Pax7^+^/Ki67^+^ cells/section, as in (E); Right panel: Histogram showing the prevalence of Pax7^+^/Ki67^+^ cells as percent of the Pax7^+^ population (n = 5, *p <0.05, ***p<0.001, unpaired student *t-*test) (J and K) Determination of cell proliferation in CTX treated muscle. *Runx1* ^*L/L*^ and *Runx1* ^*f/f*^ */Myf5*::*Cre* mice were treated with CTX and analyzed for BrdU incorporation. (I) Images of *Runx1* ^*L/L*^ and *Runx1* ^*f/f*^ */Myf5*::*Cre* gastrocnemius muscle sections stained with H&E (top) or anti-BrdU Ab (bottom). Scale bars, 50 μm. Results from one of seven *Runx1* ^*L/L*^ or *Runx1* ^*f/f*^ mice analyzed with similar findings are shown. (J) Histograms showing the average number of BrdU^+^ cells/section. BrdU^+^ cells were counted in several sections spanning 1 mm length of treated muscle. Values are mean±SE (n = 7, *p <0.05).
(TIF)

S4 Fig Loss of Runx1 does not change cell viability and leads to accelerated PM differentiation. (A-C) Parallel SC cultures from *Runx1* ^*L/L*^ mice were infected with either Ad-GFP or Ad-Cre-GFP, resulting in *Runx1* ^*L/L*^ and *Runx1* ^*-/-*^ myoblasts, respectively. Cells were grown in proliferation medium for 4 days allowing diminution of Runx1 protein level in the *Runx1* ^*-/-*^ myoblasts. (A) Average doubling time of *Runx1* ^*L/L*^ and *Runx1* ^*-/-*^ PM cultures was determined as described (n = 5, ±SD), *P<0.05). (B and C) *Runx1* ^*L/L*^ and *Runx1* ^*-/-*^ PM were stained with propidium iodide (PI) and FACS analyzed for cell cycle progression. Diagrams of cell cycle fractions of *Runx1* ^*L/L*^ PM (infected with Ad-GFP) (B) and *Runx1* ^*-/-*^ PM (infected with Ad-Cre-GFP) (C) are shown. Red and green arrows indicate increase in % G1 cells and decrease in % of S and G2/M of *Runx1* ^*-/-*^ vs. *Runx1* ^*L/L*^ cells. Results from one of four *Runx1* ^*L/L*^ or *Runx1* ^*-/-*^ PM cultures analyzed with similar findings are shown. (D-E) Cell death of *Runx1* ^*L/L*^ and *Runx1* ^*f/f*^ PM cultures were analyzed using PI staining. Proliferating PM cultures were collected, stained with PI and subjected to FACS analysis. (D) Representative histogram overlay of *Runx1* ^*L/L*^ and *Runx1* ^*f/f*^ PM cultures. Unstained WT PM (red) served as negative control, and heat permeabilized (see M&M) WT PM served as positive control. (E) Quantification of cell death rates (n = 4, average ±SD). (F) Immunostaining of PM cultures with anti- MyoD and MHC Abs and stained with DAPI as nuclear marker to record spontaneous differentiation. (I-IV) *Runx1* ^*L/L*^ and (V-VIII) *Runx1* ^*-/-*^ at x200 magnification. (IX-XII) *Runx1* ^*L/L*^ and (XIII-XVI) *Runx1* ^*-/-*^ at x630 magnification. Scale bars, 50μm and 20μm for the X200 and X630 magnifications, respectively. Results from one of four *Runx1* ^*L/L*^ or *Runx1* ^*-/-*^ cultures analyzed with similar findings are shown.
(TIF)

S5 Fig Decrease in Runx1 protein level during PM differentiation. (A) Nuclear extracts of proliferating and differentiating PM were analyzed by western blotting. Emerin was used as loading control. (Pro) proliferating PM; (Diff) PM incubated in differentiation medium for 24 or 48 h. Representative experiment out of four PM cultures from each time point with similar findings is shown. (B) Quantification of protein levels at the various differentiating stages presented in (A). Reads were calibrated according to Emerin and normalized to Runx1 protein level in proliferating PM (average ±SD), **P<0.001). (C) Western blotting of nuclear extracts from cultured proliferating myoblasts (MB) or differentiating myotubes (MT) (n = 4) as described in (A). Twelve hours prior to nuclear extraction, either vehicle (DMSO) or 5μM Bortezomib were added to the culture. Representative experiment out of four PM cultures from each treatment with similar findings is shown. (D) Quantification of Runx1 protein levels in the western blots shown in (C). (n = 4, average ±SD, *P<0.05, **P<0.001). (E) RT-qPCR documenting *Runx1* RNA level during PM differentiation. RNA isolated from proliferating and differentiating PM cultures (n = 5) at 0 and 24, 48 and 72 hours following differentiation induction was analyzed by RT-qPCR. Values shown are ±SD.
(TIF)

S6 Fig MyoD ChIP-seq analysis. MyoD ChIP-seq was performed using proliferating PM.(A) Distribution of MyoD ChIP-seq peaks relative to TSS of known genes. (B) Enriched TF motifs among MyoD-bound regions in ChIP-seq data. (C) Venn diagrams showing the overlap between Runx1 and MyoD-bound regions (*p*<1e-4, booststrap test).
(TIF)

S7 Fig c-Jun expression and ChIP- seq analysis. (A and B) Analysis of c-Jun expression during PM differentiation. (A) RT-qPCR documenting *c-Jun* RNA level during PM differentiation. RNA isolated from proliferating (Pro) and differentiating PM cultures (n = 4) at 24, 48 and 72 hours following differentiation induction was analyzed by RT-qPCR. Values shown are ±SD. (B) Nuclear extracts of proliferating (time 0) and differentiating PM incubated in differentiation medium for 24, 48 or 72 hours were analyzed by western blotting. Emerin was used as loading control. Representative experiment out of four PM cultures from each time point with similar findings is shown. (C) Enriched TF motifs among c-Jun-bound ChIP-seq regions. (D) Venn diagram showing the overlap of regions bound by Runx1 and c-Jun. (E) c-Jun binding in *Runx1* ^*L/L*^ (*Rx1* ^*L/L*^) and *Runx1* ^*f/f*^ (*Rx1* ^*f/f*^) PM was recorded using qChIP. Following c-Jun ChIP, qPCR of selected RMJ- bound loci was conducted, comparing their enrichment in the ChIP product vs. relevant input DNA. Enrichment of these loci in *Runx1* ^*L/L*^ (*Rx1* ^*L/L*^-blue) and *Runx1* ^*f/f*^ (*Rx1* ^*f/f*^-red) is presented as fold enrichment compared to input. *Cd19* locus served as a negative control. MyoD qChIP, was conducted using similar conditions, but did not yield comparable reduction in binding to these RMJ loci in *Runx1* ^*f/f*^ PM. (F) Normal *c-Jun* expression in Runx1-deficient PM. Upper panel: RT-qPCR analysis showing *c-Jun* RNA levels in *Runx1* ^*f/f*^ (*Rx1* ^*f/f*^) compared to *Runx1* ^*L/L*^ (*Rx1* ^*L/L*^) derived PM; Lower panel, Western blot of nuclear extracts from cultured *Runx1* ^*L/L*^ (*Rx1* ^*L/L*^) and *Runx1* ^*f/f*^ (*Rx1* ^*f/f*^) PM, reacted with anti- Runx1 and c-Jun Abs. Emerin served as loading control.
(TIF)

S8 Fig Expression of RMJ- regulated genes. (A) Heat maps showing expression data of WT (*Runx1* ^*L/L*^) vs. *Runx1* ^*f/f*^ differentially expressed genes that had Runx1-, MyoD- and c-Jun-bound ChIP-seq sites (up to 200kb proximal to TSS). 408 corresponding genes are presented. Microarray fluorescence intensities were normalized to the mean of each gene, and are presented as ±SD values (color-coded from -1 to +1). (B) Heat map showing log2 fold expression in *Runx1* ^*f/f*^ vs. WT (color-coded from -5 to +5) of selected Runx1-responsive genes with ChIP-seq bound Runx1, MyoD and c-Jun
(TIF)

S9 Fig Cross analysis of histone marked enhancer ChIP-seq and ATAC-seq. ChIP-seq using histone modification antibodies was performed (H3K4me1 and H3K27ac), and compared to prior TF ChIP-seq data. (A) Venn diagram showing the overlap of regions bound by Runx1 (“Runx1 ChIP”) and “active” enhancers (Enhancers histone marks ChIP). (B) Venn diagram showing the overlap of regions bound by Runx1, MyoD and c-Jun (“RMJ ChIP”) and “active” enhancers, as in (A). (C) Venn diagram showing the overlap of regions bound by Runx1 (Runx1 ChIP) and open chromatin (ATAC-Seq). ATAC sequencing was performed on WT proliferating PM and compared to prior ChIP-seq and Expression data. The common regions were cross-analyzed with the Runx1-responsive genes. (D) Venn diagram showing the overlap of regions bound by Runx1, MyoD and c-Jun (RMJ ChIP) and open chromatin, as in (C). (E-F) qChIP of histone markers characteristics to poised enhancers. PM cultures of WT proliferating (WT Pro.), or 24 post- differentiation induction (WT Diff.) or *Runx1* ^*f/f*^ proliferating (*Runx1* ^*f/f*^) were subjected to ChIP using anti H3K4me1 or H3K27me3 antibodies. qPCR of selected loci was conducted, comparing enrichment of H3K4me1 and H3K27me3 in the ChIP product vs. relevant input DNA. Enrichment of these loci is presented as fold enrichment compared to input. (E) H3K4me qChIP. (F) H3K27me3 qChIP. Values are mean±SEM (n = 3).
(TIF)

S10 Fig Validation of Runx1-dependant activation of selected regulatory elements. Dual Luciferase reporter assay was conducted using selected intact or mutated Runx1 bound regulatory elements of *Myog*, *Tnnt1*, *Myom2* and *Myh8*. The data represent means ± SD of two experiments performed in triplicates. (A) pTK-Luc plasmids, carrying regulatory elements, were transfected into HEK293 cells with either pCDNA3.1 (empty vector, blue columns) or with Runx1 expression vector (red columns). Luciferase expression was normalized to the empty vector luminescence. (B) Regulatory elements of *Myog* and *Tnnt1* were mutated to eliminate RUNX binding motifs. These elements were also cloned into pTK-Luc vectors. HEK293 cells were transfected with either intact regulatory elements (WT) or RUNX mutated regulatory elements (Mut) co-transfected with either empty vector or Runx1 expression vector, as in (A). Luciferase expression was normalized to the WT+ empty luminescence. (C) The same WT and mutant vectors were transfected into PM cells. Luciferase expression was normalized to PM transfected with pTK-Luc empty vector.
(TIF)

S1 Table Differentially expressed genes in *Runx1* ^*f/f*^ PM. Transcriptome comparison of *Runx1* ^*L/L*^ and *Runx1* ^*f/f*^ PM cultures was performed as described. Genes that were differentially expressed are presented. Verification of fold change using RT-qPCR was performed for several genes of interest.
(XLSX)

S2 Table Runx1-regulated genes in PM. Runx1 PM ChIP-seq data was cross analyzed with transcriptome data (S1 Table) as described. Genes that are differentially expressed in *Runx1* ^*f/f*^ and have a proximal Runx1 binding site are presented.
(XLSX)

S3 Table Prominent Runx1 –regulated genes. (XLSX)

S4 Table GO genes association of Runx1 binding peaks (GREAT derived). Runx1 ChIP-seq peaks that were assigned to Runx1- dependent genes were analyzed using GREAT tool. Highly enriched terms are presented.
(XLSX)

S5 Table Ingenuity Pathway Analysis of RMJ-regulated genes. Listed are top enriched IPA functions.
(XLSX)

S6 Table Runx1- responsive genes in *mdx/Runx1* ^*f/f*^. Transcriptome comparison of *mdx* and *mdx/Runx1* ^*f/f*^ soleus muscle at P60 was performed as described. Genes that were differentially expressed are presented.
(XLSX)

S7 Table *In vivo* High confidence Runx1- regulated genes. *mdx* Runx1- responsive genes were compared to PM RMJ- regulated genes. Common genes are presented. Verification of fold change using RT-qPCR was performed for several genes of interest.
(XLSX)

S8 Table Sequence of primers used in this study. (XLSX)
